# Gene-Based Modeling of Methane Oxidation in Coastal Sediments: Constraints on the Efficiency of the Microbial Methane Filter

**DOI:** 10.1021/acs.est.3c02023

**Published:** 2023-08-16

**Authors:** Wytze K. Lenstra, Niels A. G. M. van Helmond, Paula Dalcin Martins, Anna J. Wallenius, Mike S. M. Jetten, Caroline P. Slomp

**Affiliations:** † Department of Earth Sciences—Geochemistry, Utrecht University, Princetonlaan 8a, 3584 CB Utrecht, The Netherlands; ‡ Department of Microbiology, Radboud Institute for Biological and Environmental Sciences, Radboud University, Heyendaalseweg 135, 6525 AJ Nijmegen, The Netherlands

**Keywords:** microbial methane oxidation, gene-centric reactive transport modeling, greenhouse gas, sediment biogeochemistry, cell-specific methane oxidation rates, microbial growth rates

## Abstract

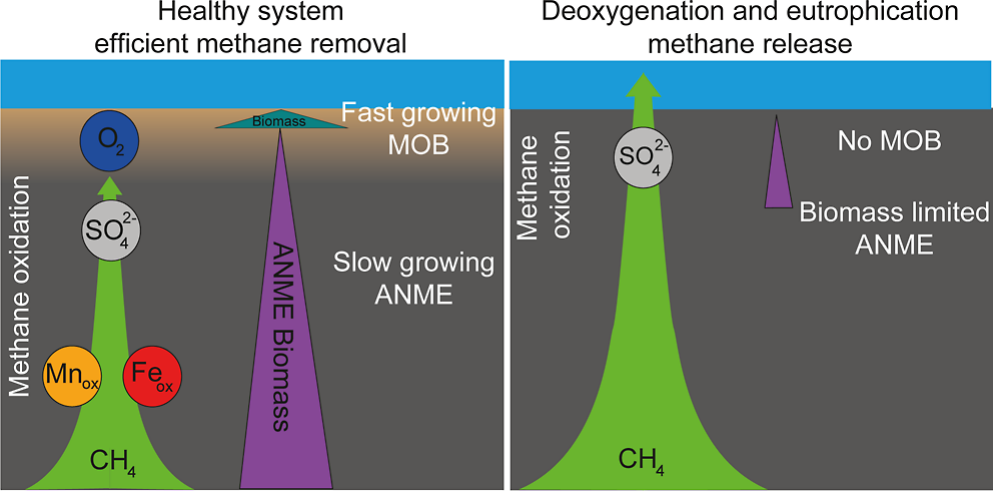

Methane is a powerful greenhouse gas that is produced in large quantities in marine sediments. Microbially mediated oxidation of methane in sediments, when in balance with methane production, prevents the release of methane to the overlying water. Here, we present a gene-based reactive transport model that includes both microbial and geochemical dynamics and use it to investigate whether the rate of growth of methane oxidizers in sediments impacts the efficiency of the microbial methane filter. We focus on iron- and methane-rich coastal sediments and, with the model, show that at our site, up to 10% of all methane removed is oxidized by iron and manganese oxides, with the remainder accounted for by oxygen and sulfate. We demonstrate that the slow growth rate of anaerobic methane-oxidizing microbes limits their ability to respond to transient perturbations, resulting in periodic benthic release of methane. Eutrophication and deoxygenation decrease the efficiency of the microbial methane filter further, thereby enhancing the role of coastal environments as a source of methane to the atmosphere.

## Abstract 2

With a novel gene-based reactive transport model, we show that the slow growth of anaerobic methane oxidizers limits methane oxidation during transient perturbations, thereby allowing the release of methane to the overlying water.

## Introduction

Methane (CH_4_) is an important greenhouse gas and its atmospheric concentration has more than doubled since the start of the industrial revolution.^1^ Methanogenesis accounts for the final step in the degradation of organic matter in marine sediments and accounts for a substantial fraction of naturally produced CH_4_.^2^ Methane emissions from the seafloor are limited, however, because most CH_4_ is converted to CO_2_ via microbially mediated anaerobic and aerobic CH_4_ oxidation.^3^ Enhanced eutrophication (i.e., enhanced nutrient input and organic matter loading) and deoxygenation can alter the balance between CH_4_ production and its oxidation, potentially resulting in high benthic CH_4_ release.^4,5^ Coastal zones are especially vulnerable to such environmental perturbations because of their relatively shallow sulfate–methane transition zone (SMTZ).^6^ It is therefore critical to better understand and quantify the effects of perturbations on marine CH_4_ dynamics and the efficiency of the microbial CH_4_ filter to constrain future CH_4_ release from marine coastal systems.

Sedimentary CH_4_ is predominantly oxidized by microbes using oxygen (O_2_) and sulfate () as electron acceptors.^3^ However, recent discoveries show that alternative anaerobic pathways such as CH_4_ oxidation coupled to Fe and Mn oxide reduction can also play a role.^7−9^ The quantitative role of metal-dependent anaerobic oxidation of CH_4_ is largely unknown. Nitrate and nitrite can also be used as electron acceptors to oxidize CH_4_,^10^ but because of their relatively low concentrations in marine sediments, they are expected to play a limited role.^11^ Microbial oxidation rates of CH_4_ coupled to different electron acceptors are often estimated via geochemical modeling or incubations with radiotracers.^12−14^ However, quantification of the in situ cell-specific rates and doubling times that ultimately control the ability of microorganisms to adapt to changing environmental conditions remains a challenge. This specifically holds for slow growing microbes, such as anaerobic methanotrophic archaea (ANME).^15,16^ As a consequence, the role of microbes in the sedimentary CH_4_ filter and their response to anthropogenic perturbations are not well understood.

Recently, reactive transport models (RTMs) that include microbial dynamics were developed to describe nitrogen dynamics in the water column of oxygen minimum zones.^17,18^ In these models, functional gene abundances were used as a proxy for the cell abundances that are associated with a given redox pathway. These studies show that the use of the functional gene approach in RTMs increases their predictive power. Here, we present a gene-based RTM for sediments, in which we use the same principles^17,18^ to investigate the controls on the microbial CH_4_ filter. We applied the model to sediments from a brackish coastal site in the Bothnian Sea that is rich in CH_4_ and Fe oxides and where both geochemical and microbial data suggest a high potential for anaerobic CH_4_ oxidation coupled to  and to Fe and Mn oxides.^19,20^ The RTM is calibrated with porewater and solid phase depth profiles and depth-dependent oxidation and reduction rates of key geochemical processes. With the RTM, we show that O_2_ and  are the key electron acceptors for CH_4_ oxidation. Metal oxides can also play an appreciable role, accounting for up to 10% of the total CH_4_ oxidized. The relatively slow growth rate of ANMEs in comparison to other microbes prevents their rapid adjustment to quickly changing environmental conditions. In dynamic systems with large temporal changes in porewater O_2_ and  concentrations, such as coastal zones, this leads to periods of high benthic CH_4_ release. With a sensitivity analysis, we investigate the response of the microbial communities to changes in environmental parameters such as bottom water O_2_ and organic matter and metal oxide deposition. We show that continued coastal eutrophication and deoxygenation will decrease the efficiency of the microbial CH_4_ filter. Ultimately, this will enhance the importance of the oxidation of CH_4_ in the water column, which is the last barrier before CH_4_ is released to the atmosphere.

## Materials and Methods

### Study Area and Sampling

**Figure 1:**
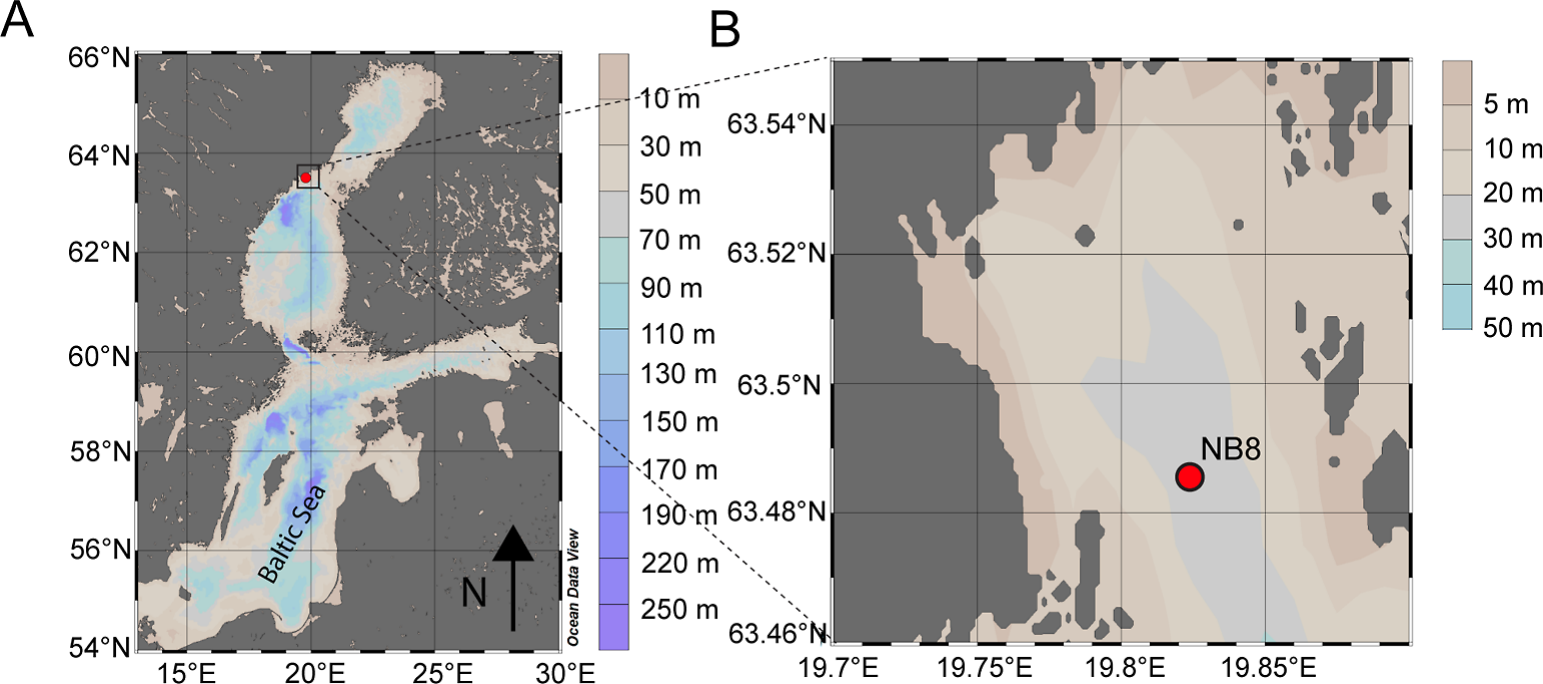
(A) Location of the Öre Estuary in the Bothnian Sea. (B) Location of sampling site NB8 in the Öre Estuary. Figure drawn using Ocean Data View.^22^

The Öre Estuary is located at the Swedish coast in the Bothnian Sea (Figure 1A). The estuary is oligotrophic and has a surface area of approximately 70 km^2^, a mean depth of 10 m, and a bottom water salinity of ca. 6. This study focuses on site NB8 that is located in the deepest part of the estuary (Figure 1B). The site is characterized by oxygenated bottom waters, bioirrigation to a depth of ca. 10 cm, and high rates of organic matter deposition (Figure SA.1).^19^ Biogeochemical processes in the Öre Estuary are strongly impacted by pulses of high Fe, Mn, and organic carbon input from the Öre River that occur every ca. 20 years and are thought to be coupled to hydrological changes on land.^19,21^ Microbial community analysis by 16S rRNA gene amplicons at our site revealed a high relative archaeal abundance of up to 90% ANMEs 2a,b.^20^ The ANMEs 2a,b become abundant in the SMTZ and follow the Fe content below the SMTZ. This indicates the potential of ANMEs 2a,b to couple CH_4_ oxidation to both  and Fe oxide reduction.

Sediment was collected during a field campaign with *R/V Botnica* in June 2019 using a Gemini gravity corer (8 cm inner diameter). In total, 11 cores were collected. Core 1 was used for porewater and solid phase analyses; core 2 was used for CH_4_ sampling; core 3 was used for O_2_ micro-profiling; cores 4–5 were used for the determination of Fe and Mn reduction and  production rates; cores 6–7 were used to determine sulfate reduction rates (SRR); cores 8–9 were used to determine CH_4_ production rates; core 10 was used to determine the sediment porosity; and core 11 was used to determine sedimentary bioirrigation rates in the sediment.

Cores for CH_4_ and  reduction rates were sampled directly after core recovery using a core liner with pre-drilled holes with a 2.5 cm depth spacing. For CH_4_, samples of 10 mL were taken with cutoff syringes from each hole and immediately transferred to a 65 mL glass bottle filled with saturated salt solution. The bottles were stoppered, capped, and stored upside down until analysis. For  reduction rates, samples of 5 mL were taken with cutoff syringes from each hole and were closed directly with parafilm.^5^

All other cores were brought back to shore for further processing. From the core for porewater and solid phase analysis, two bottom water samples were taken, and subsequently the core was transferred into intervals of 1–4 cm under a nitrogen atmosphere at bottom water temperature. Each sediment sample was sliced into a 50 mL centrifuge tube. The 50 mL centrifuge tubes were centrifuged at 4000 rpm for 20 min to extract porewater. Cores for Fe and Mn oxide reduction and  production rates were sliced under an anoxic atmosphere in 7 different intervals (0–0.5, 0.5–3.5, 3.5–6.5, 17–20, 30–33, 45–48, and 57–60 cm) into plastic beakers, except for the top sample that was sampled in a 50 mL centrifuge tube. Cores for CH_4_ production rates were sliced under an anoxic atmosphere in 6 different intervals (0–4, 9–12, 21–24, 33–36, 49–52, and 69–72) into geochemical bags. The core to determine the sediment water content was sliced into intervals of 1–2 cm into pre-weighted 50 mL greiner tubes.

### Porewater

High-resolution depth profiles of dissolved O_2_ were obtained in a separate sediment core directly after retrieval using microelectrodes (50 μm resolution) and a two-dimensional micromanipulator. Calibration was performed with a 2-point calibration with 100% oxygen-saturated and nitrogen-purged artificial seawater using the CAL300 calibration chamber (Unisense). Bottom and porewater samples were filtered through 0.45 μm pore size filters and subsampled under a nitrogen atmosphere. Subsamples were taken for analysis of , , , hydrogen sulfide (where H_2_S represents the sum of H_2_S, HS^–^, and S^2–^), dissolved Fe, and dissolved Mn. Subsamples for  and  were stored frozen at −20 °C. All other subsamples were stored at 4 °C until analysis.

Samples for  were analyzed with ion chromatography (detection limit of <75 μmol L^–1^; average analytical uncertainty based on duplicate and triplicate is 1%). For H_2_S, 0.5 mL of porewater was immediately transferred into a 4 mL glass vial containing 2 mL of a 2% zinc acetate solution to trap the H_2_S as ZnS. Sulfide was determined spectrophotometrically by the complexion of the ZnS precipitate in an acidified solution of phenylenediamine and ferric chloride.^23^ Subsamples taken for dissolved Fe and Mn were acidified with 10 μL 30% suprapur HCl per mL of sample and were analyzed by inductively coupled plasma-optical emission spectrometry (ICP-OES; PerkinElmer Avio 500). Porewater  and  concentrations were determined colorimetrically using the indophenol-blue method^24^ and with a Gallery Automated Chemistry Analyzer type,^25^ respectively. For , the standard deviation of duplicate samples was below 2%.

Samples for CH_4_ were prepared for measurement by injecting 10 mL of nitrogen headspace into the bottle. Subsequently, the CH_4_ concentrations in the headspace were determined by injection of a subsample (50–200 μL) into a Thermo Finnigan Trace GC gas chromatograph (flame ionization detector), after which CH_4_ concentrations were corrected for sediment porosity.

### Solid Phase

Sediment samples that were analyzed for porosity were dried in an oven at 60 °C, and the porosity was determined from the weight loss. Sediment that was sliced under an anoxic atmosphere was freeze-dried. The freeze-dried sediments were ground and homogenized inside an argon-filled glovebox and subsequently separated into a fraction that was stored under oxic conditions (the oxic fraction) and a fraction that was stored under a nitrogen atmosphere (the anoxic fraction). The speciation of solid phase Fe and Mn was determined on the anoxic subsamples to avoid oxidation artifacts.^26^ A subsample of circa 300 mg from the oxic fraction was decalcified with 2 wash steps of 1 M HCl^27^ and subsequently dried, powdered, and analyzed for carbon using an elemental analyzer (Fisons Instruments NA 1500 NCS). Organic C content was determined after correction for the weight loss following decalcification.

Sedimentary Fe and Mn speciation was determined on ca. 50 mg from the anoxic fraction using a 5-step sequential extraction procedure (Table SA.1) based on.^28−30^ After extraction, all solutions were filtered through 0.45 μm pore size filters prior to analysis. Total Fe and Mn in the extraction solutions were determined via ICP-OES. Both Fe(II) and total Fe were measured in the 1 M HCl solution, and Fe(III) was calculated by subtracting the Fe(II) pool from total Fe. The average analytical uncertainty for Fe and Mn is <2%. The sedimentation rate at site NB8 was determined on ^210^Pb data from a sediment core that was sampled in August 2015 and was found to be 2.75 cm yr^–1^ (Figure SA.2).

### Geochemical Rates

Fe and Mn reduction and  production rates were determined in incubations with a duration of 2 days.^31,32^ SRRs were determined on two separate sediment cores.^5,33^ The bioirrigation rate was determined in a 2 day incubation of a sediment core in which the inert tracer bromide was added to the overlying water.^34^ Methanogenesis was determined via bottle incubations.^35^ See Section SA.1 for a more detailed description of the methods for the rate determinations.

### Construction and Calibration of the Gene-Based RTM

The model that we applied to our site describes the mass balance of 9 dissolved and 8 particulate species and is a modified version of a standard multicomponent RTM based on the principles outlined by.^36^ Here, we extended this model to include the dynamics of key microbial groups that facilitate CH_4_ oxidation. We included 4 different groups of microbes that correspond to a particular metabolism:^18,37^ (1) aerobic CH_4_ oxidation; (2)  driven anaerobic oxidation of CH_4_ (–AOM); (3) Fe oxide driven anaerobic oxidation of CH_4_ (Fe–AOM); and (4) Mn oxide driven anaerobic oxidation of CH_4_ (Mn–AOM). In the model, substrate-dependent microbial growth is described using Michaelis–Menten kinetics with an optional inhibition factor,^17,37^ including the thermodynamic potential factor *F*_T_;^17^ that accounts for the Gibbs free energy available to drive the metabolism. The equation that describes modeled microbial growth in cell yr^–1^ cm^–3^ is defined as
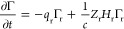
1where −*q*_r_ is the death rate (yr^–1^), Γ_r_ is the microbial abundance (cells cm^–3^), c is the average dry cell mass (gram cell^–1^), *Z*_r_ is the biomass production coefficient (gram mol^–1^), and *H*_r_ is the cell-specific reaction rate (mol yr^–1^ cell^–1^). The rate of the processes in mol yr^–1^ cm^–3^ is defined as
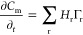
2where *C*_m_ is the concentration of the reactant. The model is calibrated with porewater and solid phase depth profiles and depth-dependent production and removal rates of key geochemical processes in the sediment. Model details and settings are given in Section SA.2.

## Results and Discussion

### Methane Dynamics in Coastal Sediments

**Figure 2:**
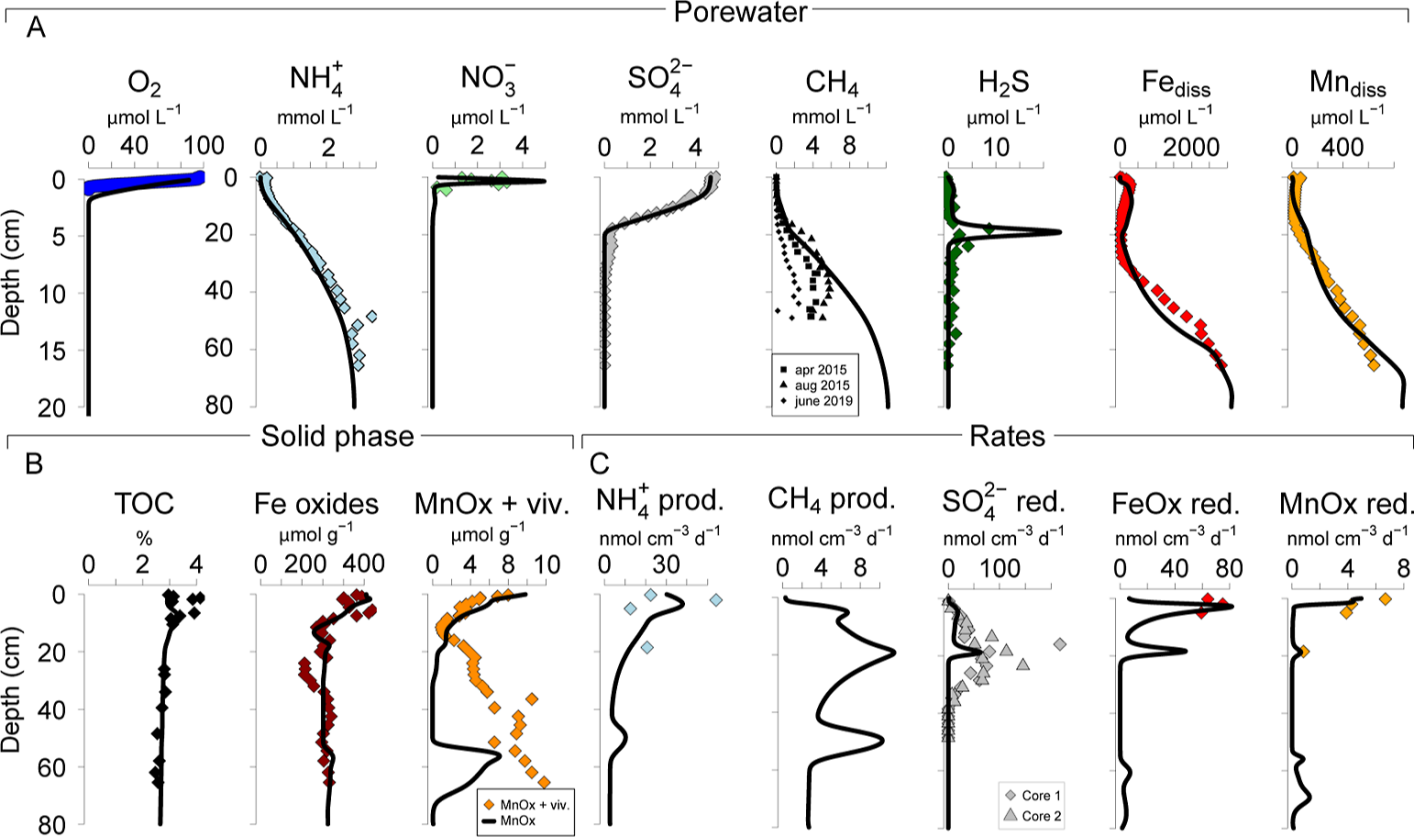
(A) Porewater depth profiles of O_2_, , , , H_2_S, dissolved Fe, and dissolved Mn; (B) solid phase depth profiles of total organic carbon, Fe oxides, and Mn oxides. Due to strong variations in the incorporation of Mn in the structure of vivianite,^38,39^ this mineral is not included in the RTM. (C) Production rates of  and CH_4_ and reduction rates of , Fe oxides (FeOx), and Mn oxides (MnOx). Colored diamonds are measured concentrations or rates, and the black lines are modeled concentrations or rates from the RTM.

At our coastal site, high rates of organic matter decomposition are evident from the limited penetration of O_2_ and nitrate () in the sediment (i.e., 0.7 and 4 cm, respectively) and high concentrations of porewater ammonium (; up to 3 mmol L^–1^; Figure 2A). Both the low salinity and active  reduction contribute to a shallow SMTZ at ca. 20 cm depth, below which CH_4_ concentrations increase up to ca. 6 mmol L^–1^. Despite high SRRs, little sulfide accumulates in the porewater because of the abundant presence of Fe oxides Figure 2.^19^ In the methanic zone, the dissolution of Fe and Mn oxides leads to high concentrations of dissolved Fe and Mn (up to 2.8 and 0.6 mmol L^–1^, respectively). This is attributed to Fe and Mn oxide-mediated oxidation of CH_4_ at depth.^19^

**Figure 3:**
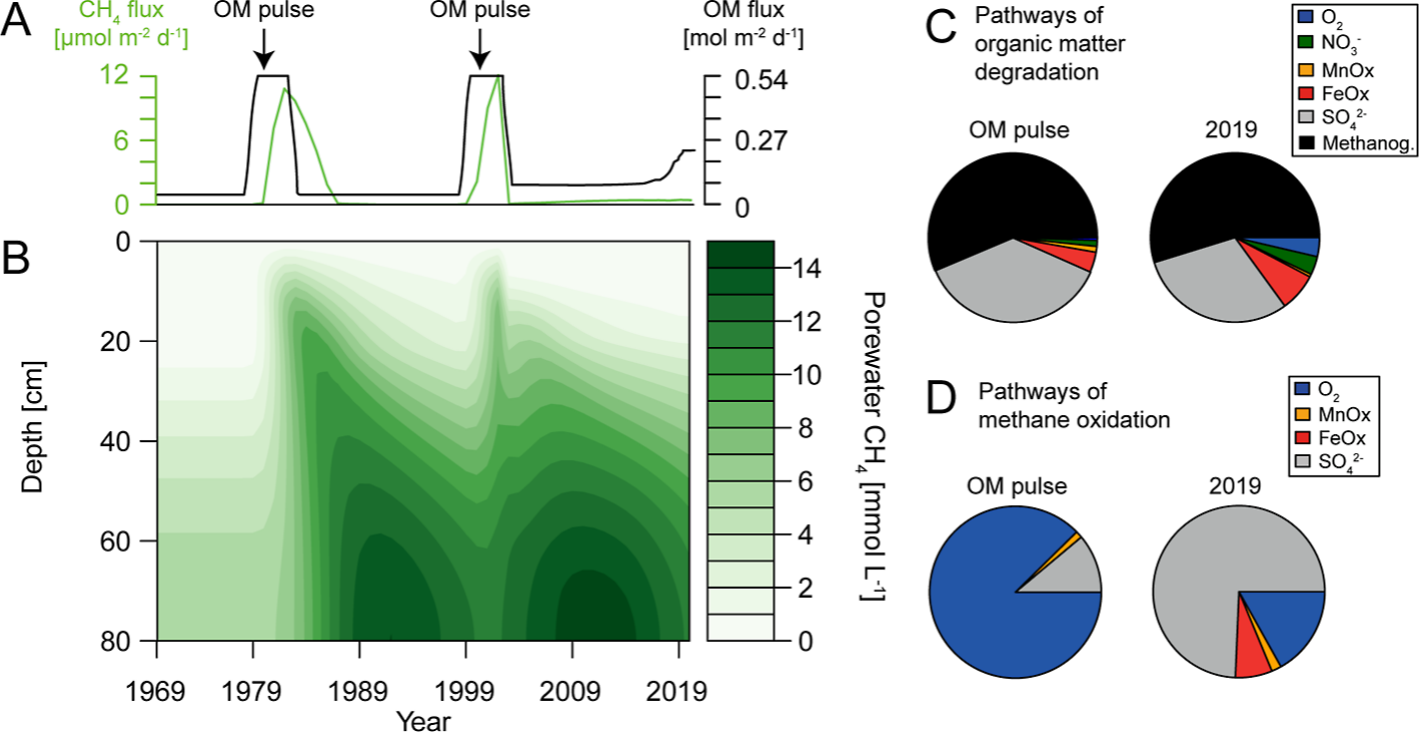
(A) Modeled transient organic matter deposition and benthic CH_4_ release; (B) heatmap of porewater CH_4_ dynamics from 1969–2019; (C,D) relative contribution of the various pathways of organic matter degradation and CH_4_ oxidation in the sediment during enhanced OM deposition and in the last year of the model run (2019), respectively.

We applied our RTM to key porewater and solid-phase depth profiles and to measured rates of CH_4_ and  production and reduction rates of , Fe oxide, and Mn oxide at our site (Figure 2). Based on previous work,^19^ we implemented a transient scenario in which a period of increased organic matter, Fe and Mn oxide deposition occurred every 20 years (Figure SA.3 and Section SA.2.3). Modeled porewater and solid-phase depth profiles adequately capture the trends in the measured profiles (Figure 2). The same holds for the modeled rates of  production and Fe and Mn oxide reduction. The modeled SRR above the SMTZ is similar to the measured rates. However, below the SMTZ, the depth profiles deviate, likely because of sample handling issues that also impact potential rates of methane production, as discussed in Section SA.3. The variations in organic matter deposition strongly impact temporal CH_4_ dynamics at our site (Figure 3A,B). After periods of enhanced organic matter deposition, methanogenesis becomes the key pathway for organic matter degradation, and porewater CH_4_ concentrations strongly increase. During these periods, microbial CH_4_ oxidation cannot keep up with the sudden increase in methanogenesis, which leads to periodic benthic CH_4_ release of up to 12 μmol m^–2^ d^–1^ (Figure 3A). This indicates that, especially in dynamic environments, such as coastal zones, benthic CH_4_ release may occur periodically because microbial abundances need to adjust to the change in CH_4_ supply and electron acceptor availability.

The various pathways of CH_4_ oxidation are highly dependent on temporal changes in organic matter deposition. In periods of enhanced organic matter deposition, O_2_ is the main electron acceptor for CH_4_ oxidation, while in the final year of our model run (i.e., 2019),  is responsible for ca. 75% of CH_4_ oxidation (Figure 3D). This is in accordance with the current understanding that the oxidation of CH_4_ in marine systems is predominantly coupled to O_2_ and .^2,3^ Recent geochemical and microbiological evidence, however, shows that Fe and Mn oxides can also mediate CH_4_ oxidation,^7,8,40^ but the quantitative importance of Fe- and Mn–AOM is largely unknown. Measured and modeled rates in North Sea and Bothnian Sea sediments suggest that Fe–AOM accounted for ca. 2–3% of the total anaerobic oxidation of CH_4_.^8,41^ In our model for metal oxide-rich sediment, Fe and Mn oxide-mediated CH_4_ oxidation is responsible for ca. 10% of the total CH_4_ oxidation in the year of sampling. This suggests that in Fe and Mn oxide-rich sediments, Fe- and Mn–AOM are able to account for an appreciable fraction of the oxidation of sedimentary CH_4_. This is likely especially important in sediments close to river mouths where the Fe and Mn oxide input is high,^42^ and the depth of the SMTZ is located relatively close to the sediment-water interface because of a low salinity.

### Abundance and Growth of Methanotrophs

**Figure 4:**
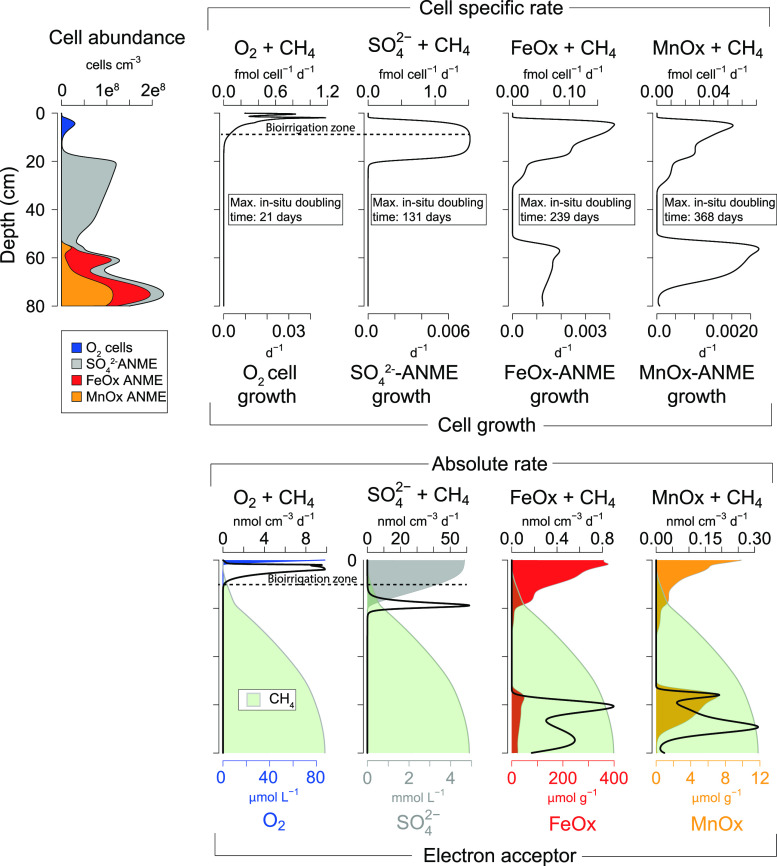
Top row: depth profiles of the cell abundance of microbes associated with CH_4_ oxidation coupled to reduction of O_2_, , Fe oxides, and Mn oxides, cell-specific rate for each pathway (fmol cell^–1^ d^–1^), and microbial community growth (d^–1^). The maximum in situ doubling time in the sediment indicates the doubling time in the last timestep of the model run. Fastest doubling times possible for O_2_ cells, –ANME, FeOX–ANME, and Mn–ANME are <1, 124, 203, and 163 days, respectively (Table SA.7). Bottom row: absolute rates of CH_4_ oxidation (nmol cm^–3^ d^–1^) with depth profiles of O_2_, , Fe oxides, and Mn oxides and CH_4_ concentration (green). Maximum concentration of CH_4_ is ca. 12 mmol L^–1^. Plots show model data of the last timestep in the RTM (i.e., for 2019).

In our RTM results, three distinct zones of cell abundance in the sediment can be distinguished based on the modeled presence of microbes involved in CH_4_ oxidation (Figure 4). Aerobic CH_4_ oxidizers are most abundant in the upper 10 cm of the sediment, while anaerobic CH_4_ oxidizer abundances are low. Below 10 cm depth in the SMTZ, cell abundances of ANMEs strongly increase. Cell abundances of ANMEs in CH_4_-rich sediments along continental margins can vary over orders of magnitude and depend on the SMTZ depth. For example, at a site with an extremely shallow SMTZ offshore Oregon (ca. 3 cm), an ANME abundance of 0.7 * 10^10^ cells cm^–3^ is reported.^43^ This contrasts with observations for a North Sea site with a deeper SMTZ (ca. 70 cm), where an abundance of ca. 4 * 10^6^ cells cm^–3^ was found.^41^ At our site, the ANMEs are almost absent above the SMTZ and become abundant in the SMTZ, where cell abundances reach ca. 1.25 * 10^8^ cells cm^–3^ at 17 cm depth (Figure 4). Below the SMTZ, the highest cell abundances (i.e., 2.2 * 10^8^ cells cm^–3^) are found at the depth where Fe oxides and Mn oxides are present. This is in accordance with the observed 16S rRNA data for our site, where ANMEs 2a,b are absent above the SMTZ but are abundant in the SMTZ and in zones where Fe oxides are present.^20^

The doubling time of microbes predominantly determines how fast microbes can adjust to varying environmental conditions in the sediment. Estimated growth rates of aerobic CH_4_ oxidizing bacteria are in the order of 12 h to days^44^ and are therefore expected to adapt quickly to variations in the availability of O_2_. The doubling time of ANMEs is not well known but has been estimated to be in the order of months.^3,16,44^ In our model scenario, the maximum doubling times of –ANME, FeOx–ANME, and MnOx–ANME are 124, 203, and 163 days, respectively (Table SA.7). The in situ doubling times in the last time step of the model are especially low for FeOx–ANME and MnOx–ANME (240 and 370 days, respectively), which indicates that their growth is limited by the presence of electron acceptors at this timepoint. The growth of –ANME is relatively fast (i.e., 130 days). This indicates that ANMEs that couple metal oxide reduction to CH_4_ oxidation grow at a slower rate than –ANMEs and therefore only become important deeper in the sediment when the microbial community has had sufficient time to grow and accumulate enough biomass. To investigate the impact of the maximum growth and death rate on microbial abundances and geochemical depth profiles, we carried out a sensitivity analysis where we multiplied the growth and death rate by a discrete factor (0.5; 0.75; 0.9; 1.1; 1.25; and 1.5; Figures SA.4 and SA.5) and assessed the changes in the profiles. We find that the model is very sensitive to changes in the growth rate and less so for the death rate, as further discussed in Section SA.4.

In sediments where geochemical processes are in a steady state,  is quantitatively the most important sink for CH_4_.^6^ However, in highly transient environments, such as coastal zones, the slow adaptation of ANMEs to transient geochemical processes can alter the role of CH_4_ oxidation by . This has previously been explored in a model study for continental margin sediments subject to increased upward advective flow of CH_4_. In the corresponding model scenario, it took >60 years for ANMEs to achieve equilibrium with the new porewater concentrations.^4^ Sulfate-reducing bacteria (SRB) can grow much faster doubling time of <1 day;^45^ than ANMEs and will typically outcompete ANME-associated SRB. Therefore, heterotrophic SRB will adapt faster to transient situations and are expected to play a key role in determining variations in SMTZ depth. This can have major implications for the role of  as an electron acceptor in CH_4_ oxidation and the efficiency of the sedimentary CH_4_ filter, as we will show in the example discussed below.

**Figure 5:**
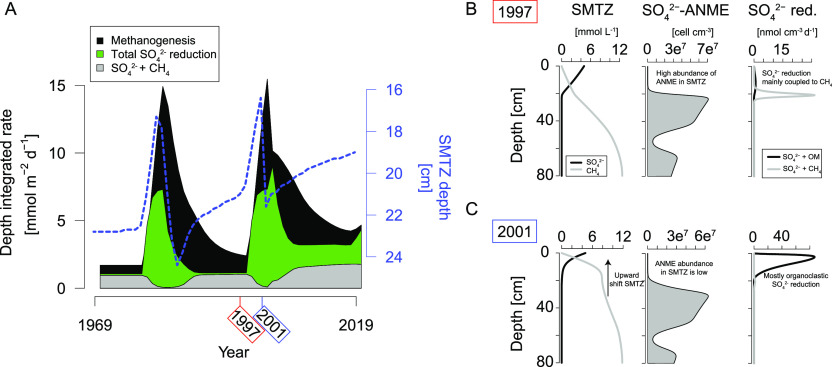
(A) Integrated rates of methanogenesis (black), total  reduction (green), and –AOM (gray) as calculated by the RTM. The blue dashed line indicates the depth of the SMZT, calculated at the first depth where the  concentration is below 0.1 mmol L^–1^ within the RTM. (B,C) show depth profiles of  and CH_4_ (mmol L^–1^), the abundance of –ANME, and the rate of  reduction coupled to oxidation of organic matter and CH_4_ for the years 1997 and 2001, respectively.

During periods of enhanced organic matter deposition in our model scenario, the SMTZ moved upward from ca. 24 to 17 cm (Figure 5A). This upward shift of the SMTZ is coupled to enhanced heterotrophic  reduction. After this shift of the SMTZ, -ANMEs that are present at the former SMTZ depth (i.e., 24 cm) no longer have access to  (Figure 5). Therefore, nearly all  reduction becomes coupled to organic matter degradation, and –AOM becomes a negligible process. This illustrates that ANMEs, because of their slow growth rate, cannot adjust quickly to a change in the availability of substrate. In our model scenario, aerobic CH_4_ oxidation then becomes the key pathway of CH_4_ oxidation (Figure 3D).

Increased organic matter input can lead to an upward shift of the SMTZ as a result of increased rates of  reduction and/or methanogenesis.^11,46,47^ Our model results suggest that it is unlikely that ANMEs facilitate a rapid upward shift of the SMTZ through –AOM because their slow growth rate hinders a quick adjustment of their biomass to varying CH_4_ and  concentrations. Therefore, a sudden upward shift of the SMTZ is likely regulated by enhanced heterotrophic/organoclastic -reduction. This would lead to the depletion of  in the zone where ANMEs are present and therefore a limited contribution of –AOM until ANMEs have had enough time to readjust to the new environmental conditions.

### Rates of CH_4_ Oxidation

Cell-specific rates of microbes (fmol cell^–1^ d^–1^) determine how much substrate microbes can use per time unit. For slow-growing microbes with low energy yields such as ANMEs,^15,16^ these cell-specific rates are largely unknown.^3,44^ Cell-specific rates can be determined during long-term or pure-culture incubation experiments. However, rates from laboratory experiments are typically orders of magnitude higher than in situ rates^7,13,48^ because of changes in, for example, substrate availability and sediment handling. Hence, they should be considered as potential rates.^2^ Quantification of the role of microbes in CH_4_ oxidation requires insight into their in situ cell-specific rates in order to couple cell abundances to absolute rates in the sediment. Our model allows us to quantify such in situ cell-specific rates for various microbes and show how these vary with sediment depth.

In our model, cell-specific rates depend strongly on substrate availability and follow Michaelis–Menten kinetics (Figure SA.6). The highest cell-specific rates are observed for aerobic methanotrophs in the zone where oxygen is pumped into the sediment via bioirrigation at 2 cm depth. Here, neither O_2_ nor CH_4_ is limiting. Cell-specific rates of –ANME are highest around the SMTZ and reach a value of ca. 1.5 fmol cell^–1^ d^–1^, which falls within the range of rates suggested in the literature of 0.2–10 fmol cell^–1^ d^–1^.^11,49^ Cell-specific rates of FeOx- and MnOx–ANME are highest in the zones where the respective metal oxides are present. Cell-specific rates are, however, 1 or 2 orders of magnitude lower compared to those of aerobic methanotrophs and –ANME, despite the fact that Fe and Mn oxides are more energetically favorable electron acceptors compared to . This is likely the case because  is a solute and therefore more easily available to microbes than solids such as Fe and Mn oxides.^7^ This additionally leads to a slower growth rate for FeOx- and MnOx–ANMEs.

The absolute rate of CH_4_ oxidation depends on both the cell-specific rate and the microbial abundance. For aerobic methanotrophs, the rate is highest in the bioirrigation zone (up to 10 nmol cm^–3^ d^–1^) and is near zero in the zone where O_2_ penetrates because of CH_4_ limitation (Figure 4). This shows that enhanced oxygenation of the sediment due to bioirrigation can be an efficient barrier for upward diffusing CH_4_ and act as an important control on benthic CH_4_ emissions. Rates of –AOM are strongly enhanced in a shallow zone of the SMTZ with rates up to 60 nmol cm^–3^ d^–1^ and are low above and below the SMTZ because of substrate limitation. Absolute rates of Fe- and Mn–AOM are only high below the SMTZ (up to 1 and 0.3 nmol cm^–3^ d^–1^, respectively; Figure 4). Rates for Fe–AOM are in the same range as found for sediments that were incubated with ferrihydrite 1–5 nmol cm^–3^ d^–1^.^41^ In situ rates of Mn–AOM are largely unknown. However, in incubation studies, very high rates were observed, i.e., ca. 40 nmol cm^–3^ d^–1^;^7,40^ when compared to those in our model. This might be because of the strongly enhanced Mn oxide concentrations in the incubations compared to the lower contents (<10 μmol g^–1^ Mn oxide) in our sediments. Despite the abundant presence of Fe and Mn oxides above the SMTZ, absolute rates are low because of the low abundance of the responsible microbes.

### Environmental Constraints on CH_4_ Oxidation

Eutrophication and deoxygenation are impacting many coastal ecosystems and have the potential to greatly alter CH_4_ dynamics in sediments.^50−52^ Enhanced eutrophication can stimulate methanogenesis, while deoxygenation can lead to less efficient CH_4_ oxidation in the sediment. The efficiency of CH_4_ oxidation is also very sensitive to other environmental perturbations, such as sea level rise and changes in precipitation, which can alter bottom water salinity^53,54^ and variations in riverine fluxes of metals, which can alter the metal oxide deposition.^55^ To investigate the effect of these perturbations on the efficiency of the sedimentary CH_4_ filter and the microbial dynamics, we carried out a sensitivity analysis where we changed the (1) bottom water salinity; (2) bottom water O_2_; (3) organic matter input; and (4) Fe and Mn oxide input.

**Figure 6:**
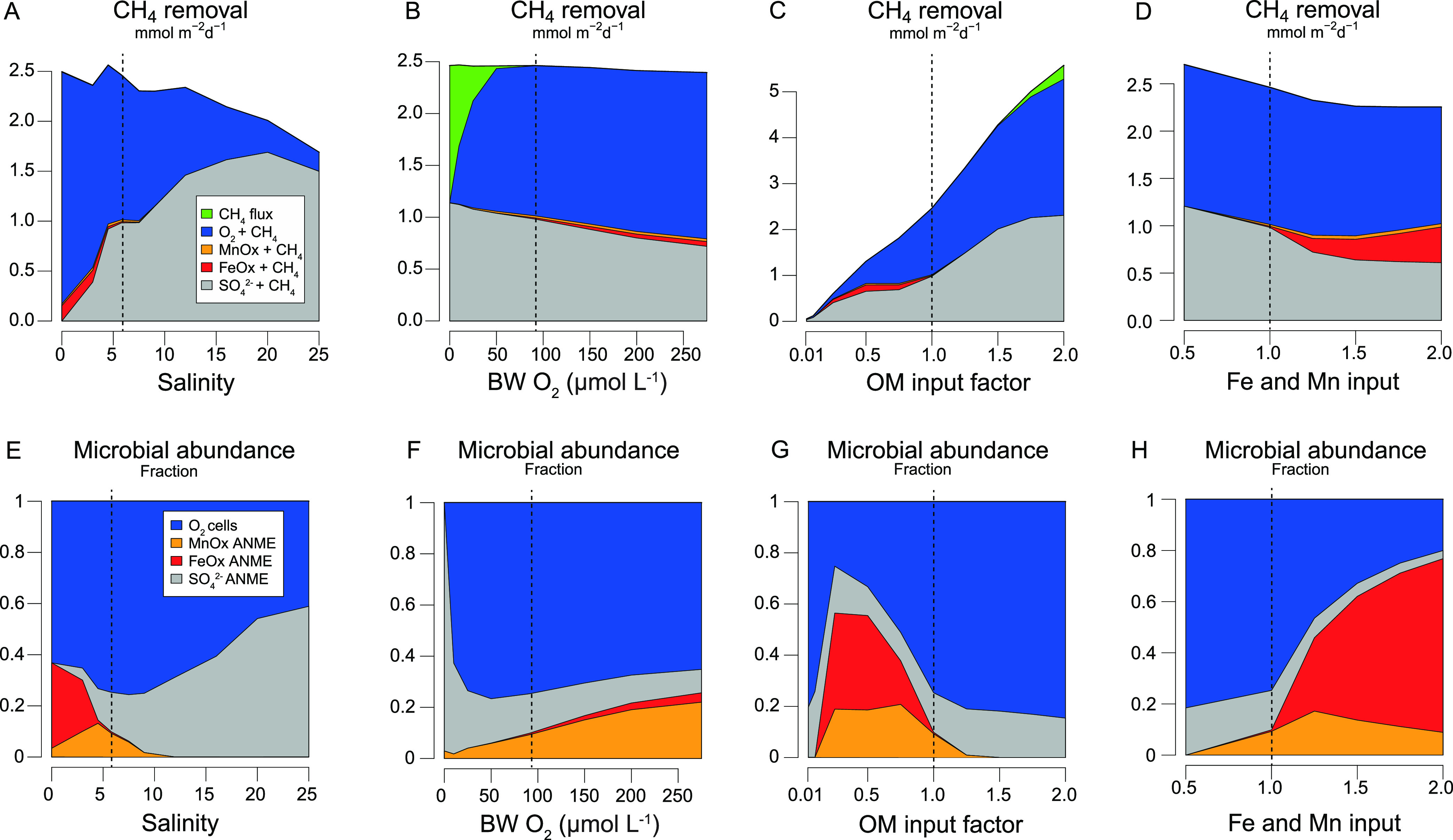
Sensitivity analysis of CH_4_ removal via benthic release (CH_4_ flux), and depth integrated oxidation rates coupled to reduction of O_2_, Mn oxides (MnOx), Fe oxide (FeOx), and  (mmol m^–2^ d^–1^) for (A) salinity; (B) bottom water O_2_; (C) the organic matter input factor; and (D) Fe and Mn oxide input factor compared to the baseline scenario. (E–H) Show the corresponding relative abundance in microbial communities for the same sensitivity analysis as A–D. The results of the baseline scenario are indicated by the vertical dashed line. The benthic flux and integrated rates are averages for the last 50 years of the transient scenario (Figure SA.3). Porewater profiles for the last time steps of the sensitivity analysis are shown in Figure SA.7.

At higher salinity, methanogenesis is suppressed because of enhanced organoclastic  reduction and -AOM (Figure 6A). Our sensitivity analysis suggests that CH_4_ oxidation is efficient over the full salinity range because of efficient CH_4_ oxidation by O_2_ and partly by metal oxides at lower salinity. However, a small benthic CH_4_ flux of 4 μmol m^–2^ d^–1^ is observed at a salinity of 0. When the bottom water O_2_ is increased, methanogenesis slightly decreases and the role of Fe- and Mn–AOM increases (Figure 6B). At lower bottom water O_2_, the importance of –AOM increases. However, when bottom water O_2_ becomes lower than 50 μmol L^–1^ oxidation of CH_4_ is not efficient enough, and enhanced benthic CH_4_ release is observed. This flux is potentially even higher when both the salinity and O_2_ would decrease.

Upon enhanced organic matter input, methanogenesis strongly increases, and the oxidation of CH_4_ coupled to O_2_ and  is enhanced (Figure 6C). However, when the deposition of organic matter increases >5%, the efficiency of CH_4_ oxidation declines and benthic CH_4_ release increases. When the deposition of Fe and Mn oxides increases, methanogenesis slightly decreases, and the role of Fe- and Mn–AOM increases (Figure 6D). However, O_2_ and  remain the major electron acceptors for CH_4_ oxidation.

The microbial composition of the aerobic and anaerobic CH_4_ oxidizing microbes in the model strongly varies (Figure 6E–H). We show that the microbial composition does not necessarily reflect the importance of a related microbially driven process. For example, FeOx- and MnOx–ANME can account for a high biomass; however, because of their relatively low cell-specific rates compared to –AOM and especially aerobic CH_4_ oxidation, the relative importance of Fe- and Mn–AOM remains limited. This highlights the importance of combining the microbial abundances as a proxy for a certain process with the cell-specific rates of the microbes.

Our modeling results suggest that the anaerobic oxidation of CH_4_ coupled to Fe and Mn oxide reduction is promoted by the following factors: (1) a low bottom water salinity, since –AOM is low and sulfide production is limited, resulting in a higher availability of Fe and Mn oxides; (2) high bottom water O_2_, since enhanced recycling of Fe and Mn increases the sedimentary Fe and Mn oxide content and there is little escape of dissolved Fe and Mn from the sediment; (3) intermediate rates of organic matter deposition, since at low organic matter deposition Fe- and Mn–AOM is limited by CH_4_ production, while at high organic matter input the availability of Fe and Mn oxides decreases because of enhanced sulfide production and subsequent Fe and Mn oxide dissolution and FeS_*x*_ precipitation; (4) a high input of Fe and Mn oxides since this directly promotes Fe- and Mn–-AOM.

## Perspectives

Microbial dynamics strongly determine geochemical processes such as CH_4_ oxidation in the sediment. Gene-centric modeling, as applied here, is an effective tool to determine the characteristics of slow-growing microbes such as anaerobic CH_4_ oxidizers and the impact of their activity on the efficiency of the microbial CH_4_ filter. The incorporation of microbial dynamics in biogeochemical models, as done here for sediments, allows us to investigate key characteristics of microbial communities. Importantly, the inclusion of microbial dynamics in RTMs is especially relevant when assessing the effects of environmental perturbations in systems where slow-growing microbes, such as ANMEs but also anammox bacteria, are involved in critical removal processes. Further improvement of the predictive power of these types of biogeochemical models can be achieved through: (1) a better quantification of microbial abundances either through qPCR to determine the amount of genes or through single-cell methods (such as CARD-FISH, nanoSIMS, or flow cytometry) or very high throughput sequencing/transcriptomics to determine the amount of active cells in the sediment; (2) a better quantification of the half rate constants and maximum cell-specific rates to further constrain the substrate dependent reaction rates (i.e., Michaelis Menten kinetics); (3) the determination of maximum growth rates of microorganisms through incubation studies; (4) the determination of death rates and the key factors that control the death rate of microorganisms; (5) the evaluation of possible inhibition factors on the growth and efficiency of CH_4_ oxidizing microbes, for example, sulfide inhibition, possibly by incubation studies.

## Supporting Information Available

Detailed description of rate measurements and model code. Figures: results of bromide tracer incubations at station NB8; measured and modeled depth profiles of ^210^Pb and porosity at station NB8; modeled transient fluxes of organic matter, Fe oxides, and Mn oxides at the sediment–water interface; model sensitivity analysis; modeled Michaelis−Menten kinetics of methane oxidation rates; porewater profiles for the sensitivity analysis; Tables: sequential extraction procedure for Fe and Mn; reaction pathways and stoichiometries implemented in the model; environmental parameters; boundary conditions of solids and solutes at the sediment−water interface; reaction equations; and potential CH_4_ production rates determined for site NB8 (PDF)
Porewater, solids phase and rate depth profiles (ZIP)

## Notes

All data needed to evaluate the conclusions in the paper are present in the paper and/or the Supporting Information.
