# Supplementary material for: Gene-Based Modeling of Methane Oxidation in Coastal Sediments: Constraints on the Efficiency of the Microbial Methane Filter
Source: Environ Sci Technol. 2023 Aug 16;57(34):12722–31. doi: 10.1021/acs.est.3c02023 (PMC10469488; doi:10.1021/acs.est.3c02023)
Supplement: Supplementary file 1 — es3c02023_si_001.pdf [file es3c02023_si_001.pdf]

Supplements: Gene-based modeling of methane oxidation in coastal sediments: constraints on the efficiency of the microbial methane filter

Wytze K. Lenstra[a,b,*], Niels A.G.M. van Helmond[a,b], Paula Dalcin Martins[b,c], Anna J. Wallenius[b], Mike S.M. Jetten[b], and Caroline P. Slomp[a,b]

[a]Department of Earth Sciences –Geochemistry, Utrecht University, Princetonlaan 8a, 3584 CB Utrecht, The Netherlands
[b]Department of Microbiology, Radboud Institute for Biological and Environmental Sciences, Radboud University, Heyendaalseweg 135, 6525 AJ Nijmegen, The Netherlands
[c]Now at Microbial Ecology Cluster, GELIFES, University of Groningen, Broerstraat 5, 9712 CP Groningen, The Netherlands

August 2, 2023

A Supplements

A.1 Rates

A.1.1 Sulfate reduction rates

Sulfate reduction rates (SRR) were measured in two cores by extracting 5 mL of wet sediment sample from predrilled and taped cores directly upon retrieval using cut off syringes following *Egger et al.* [2016b]. Within 72 hours of core retrieval, 100 μL of carrier free $^{35}SO_4{}^{2-}$ (91 kBq) was injected in the syringes. The sediment was incubated for 24 h in the dark under a nitrogen atmosphere, after which it was transferred to 50 mL centrifuge tubes containing 20 mL of deoxygenated 20% zinc acetate to precipitate dissolved H_2S and inhibit biological activity [*Fossing and Jørgensen*, 1989; *Kallmeyer et al.*, 2004]. Upon analysis, samples were rinsed twice using deoxygenated bottom water (10 mL) and centrifuged for removal of unreacted $^{35}SO_4{}^{2-}$ [*Egger et al.*, 2016b]. The reduced S was extracted with an acidic chrome chloride solution (48 h) via the passive diffusion method [*Burton et al.*, 2008]. Sulfate

reduction rates were calculated by comparing the activity (decays per minute) of the radiolabeled total reduced inorganic sulfur (aTRIS) to the total SO_4^{2-} (aTOT) radiotracer [*Kallmeyer et al.*, 2004].

A.1.2 Fe and Mn reduction and NH_4^+ production rates

Two cores were sliced under a nitrogen atmosphere for 6 sediment depth intervals of 3 cm (0.5-3.5, 3.5-6.5, 10-13, 20-23, 30-33, 40-43 cm) into beakers in which the sediment was subsequently homogenized. Additionally, the surface layer 0-0.5 cm was sampled in a 50 ml greiner. Samples from the beakers were taken at ca. t = 0, 8, 20, 30 and 48 hours under a nitrogen atmosphere. For the top sediment, samples were only taken at t=0 and t=48 because of the limited amount of material. During sampling, approximately 10 ml of sediment from each beaker was transferred into a 50 ml greiner tube, which was centrifuged at 4500 rpm for 10 minutes to extract porewater. Subsequently the porewater was filtered over 0.45 μm and subsampled for ICP-OES (2 mL, acidified with 20 μL 35% suprapur HCl) and NH_4 (remainder of sample). ICP-OES samples were stored at 4°C, NH_4^+ samples were stored at -20°C. All beakers and the greiner were stored under a nitrogen atmosphere at in-situ temperature during incubation. Dissolved Fe, Mn and NH_4^+ concentrations were determined in the same way as porewater samples. Fe and Mn reduction and NH_4^+ production rates were determined by fitting a linear regression to the concentration time series for each beaker/greiner. Only regressions with R^2 >0.5 were considered.

A.1.3 CH_4 production rates

Sediments for incubations to measure potential CH_4 production rates were sliced into intervals of 3 to 4 cm (0-4, 9-12, 21-24, 33-36, 49-52 and 69-72 cm) under a nitrogen atmosphere. The slices were stored anoxically in the dark at 4°C for one month from sample collection until bottles were assembled. For this, 5 g of wet sediments was placed in 60 ml-serum bottles, and sulfate-free artificial seawater (ASW) medium pH 7.5 was added to create a 1:1 diluted slurry. The ASW medium was adapted from that of [*Kester et al.*, 1967] to achieve a salinity of 5.3. No trace elements or vitamins were added. Methane production was monitored via injection of 100 μl-headspace samples into an HP 5890 gas chromatograph with a detection limit of <1 ppm. Each gas sample was measured in triplicate and

results were averaged.

A.1.4 Bromide incubations

A sediment core was incubated with the inert tracer bromide to determine bioirrigation rates [*Martin and Banta*, 1992]. Directly after core retrieval, the volume of the overlying water was adjusted to ca. 500 mL and a concentrated bromide solution was added to the overlying water to achieve a concentration of ca. 3 $mmol\ L^{-1}$ following [*Lenstra et al.*, 2019]. During a two-day incubation at in-situ temperature, the overlying water was kept saturated with O_2 and well-mixed by bubbling with air. After incubation, the top 20 cm of sediment was sliced into intervals of 0.5 to 2 cm and centrifuged for 20 min at 4500 rpm. After centrifugation, the supernatant was filtered (0.45 μm) and stored at 4°C. Bromide concentrations were determined with ion chromatography. Subsequently porewater bromide was modeled using a 1-D, nonlocal exchange function [*Emerson et al.*, 1984; *Boudreau*, 1984]. Here, dissolved bromide is described as (Eq. A.1),

$$\frac{\partial(\varphi c)}{\partial t} = \frac{\partial}{\partial z}\left(\varphi D_s \frac{\partial c}{\partial z}\right) - \varphi\alpha(c - c_0) \tag{A.1}$$

where φ is the depth dependent sediment porosity, z is depth in the sediment (cm) and D_s is the sediment diffusion coefficient for bromide. The molecular diffusion coefficient D_s at site NB8 was corrected for tortuosity in the porous medium [*Boudreau*, 1996a] and for the ambient salinity S, temperature T (Table A.4) and pressure using the R package CRAN: marelac [*Soetaert et al.*, 2010], which implements the constitutive relations listed in [*Boudreau*, 1997]. α is the nonlocal bioirrigation function for bromide, which is assumed to be time-invariant. c is the concentration of bromide in the porewater and c_0 is the bromide concentration of the overlying water. In the model, porosity is interpolated linearly for every depth layer in the model (1 layer every mm) between measured points. The bioirrigation function (α) was determined by fitting porewater bromide depth profiles after incubation to equation A.1.

A.2 Reactive transport model

General model description

The reactions in the reactive transport model describe organic matter degradation coupled to different electron acceptors. Reactions are divided in primary redox reactions and other biogeochemical reactions (Table A.6). The succession of oxidants during organic matter degradation [*Froelich et al.*, 1979] is described by means of Monod kinetics (Table A.3; *Boudreau* [1997]). This means that the oxidants with the highest metabolic free energy yield are preferentially used until they become limiting and the oxidant with the next highest energy yield is used [*Berg et al.*, 2003; *Boudreau*, 1996b; *Wang and Van Cappellen*, 1996]. Respiratory reactions occur where O_2, NO_3^-, MnO_2, $Fe(OH)_3$ and SO_4^{2-} serve as electron acceptors, and finally, organic matter is subject to methanogenesis (*Lenstra et al.* [2018]; Table A.3). Organic matter includes carbon (C) and nitrogen (N) in a C:N ratio of 7.1:1 (Table A.4). In the model, oxidation of CH_4 is possible with O_2, NO_3^-, MnO_2, $Fe(OH)_3$ and SO_4^{2-}. Due to strong variations in the incorporation of Mn and other cations in the structure of vivianite [*Rothe et al.*, 2016; *Kubeneck et al.*, 2021], this mineral is not included in the model. Dissolved inorganic carbon in the model is calculated as the sum of the carbon in CO_2 and HCO_3^{2-}, which is produced or consumed by modeled reactions (Table A.6).

The generic mass conservation equations for solids and solutes are described by Eq. A.2 and A.3;

$$(1-\phi)\frac{\partial C_s}{\partial t} = -(1-\phi)v\frac{\partial C_s}{\partial z} + \sum R_s \tag{A.2}$$

$$\phi\frac{\partial C_{aq}}{\partial t} = \phi D'\frac{\partial^2 C_{aq}}{\partial z^2} - \phi u\frac{\partial C_{aq}}{\partial z} + \sum R_{aq} \tag{A.3}$$

$$D' = \frac{D_m}{1 - ln\phi^2} \tag{A.4}$$

where C_s is the concentration of solid species ($mol\ L^{-1}$), C_{aq} is the concentration of dissolved species ($mol\ L^{-1}$), t is time (yr), ϕ is the sediment porosity, v and u are the advective velocities of solid and dissolved species ($cm\ yr^{-1}$), respectively. Variables v and u were described by a depth-dependent

function to account for changes in porosity [*Meysman et al.*, 2005]. Distance from the sediment-water interface is z (cm), D' is the diffusion coefficient of dissolved species ($cm^2\ yr^{-1}$), corrected for tortuosity in the porous medium [Eq. A.4 *Boudreau*, 1996c]. $\sum R_s$ and $\sum R_{aq}$ are the net reaction rates from the chemical reaction (Table A.3) for solid and dissolved species.

Porosity (ϕ) is described by Eq. A.5 to account for sediment compaction [*Meysman et al.*, 2005; *Reed et al.*, 2011b],

$$\phi(x) = \phi_\infty + (\phi_0 - \phi_\infty)e^{-\frac{x}{y}} \tag{A.5}$$

where ϕ_0 is the porosity at the sediment-water interface, ϕ_∞ is the porosity at depth and y is the porosity attenuation factor/e-folding distance (Table A.4). The model code was written in R with the use of the marelac geochemical dataset package [*Soetaert et al.*, 2010]. To calculate the transport in porous media, the R package Reactran was used [*Soetaert and Meysman*, 2012]. The set of ordinary differential equations was solved numerically with the Lsode integrator algorithm [*Petzold*, 1983]. Zero gradient boundary conditions were applied to the base of the model domain for all chemical species. The total depth of the model was set to 80 cm (divided into 800 grid cells of 0.1 cm). Reaction parameters were mostly taken from literature or obtained within existing parameter ranges (Table A.7). If these were not available, or no fit to the data could be obtained with existing ranges, parameters were constrained by fitting the model to the measured data.

A.2.1 Microbial modeling and equations

The model considers 4 different microbial groups that couple the oxidation of CH_4 to O_2, ${SO_4}^{2-}$, Fe oxides and Mn oxides, respectively (Table A.6: R27-R32). Several archaea have been shown to mediate the anaerobic oxidation of CH_4 (AOM), including the ANME-1, ANME-2a-c, Methanoperedenaceae, and the ANME-3 [*Leu et al.*, 2020]. It has been suggested that archaea can switch oxidation pathways from ${SO_4}^{2-}$ to for example Fe and Mn oxides, but this does not hold for all groups. For example ANME-1 can perform S-AOM [*Boetius et al.*, 2000] but is likely not involved in Fe-AOM [*Aromokeye et al.*, 2020]. In the model, we therefore decided to include 4 different groups of mi-

crobes that correspond to a particular metabolisms without accounting for switches between different metabolisms. We also did not include cells that facilitate the oxidation of CH_4 coupled to NO_x because this is likely a negligible pathway in marine sediments [*Jørgensen*, 2021]. The production of cells at any depth is driven by the release of energy from their reactions, and is proportional to the Gibbs free energy multiplied by the reaction rate. The cell specific reaction rate ($mol\ yr^{-1}\ cell^{-1}$) in given as follows,

$$H_r(C) = V_r F_T \times \frac{C_m}{K_{rm} + C_m} \times \frac{K_{rn}}{K_{rn} + C_n} \tag{A.6}$$

where, V_r is the maximum gene-specific rate ($mol\ cell^{-1}\ yr^{-1}$; Table A.7), F_t is the thermodynamic potential factor (unitless) following *Reed et al.* [2014]; *Louca et al.* [2016] (Eq. A.9), K_{rm} is the half-saturation constant of the substrate, K_{rn} is the half-inhibition constant and C_n is the concentration of the inhibitor. Following *Reed et al.* [2014]; *Louca et al.* [2016], we calculated the biomass production coefficient ($g\ mol^{-1}$) as

$$Z_r = 2.08 \times \gamma_r^e - 0.0211 \Delta G_r \tag{A.7}$$

where, γ_r^e is the negative stoichiometric coefficient and ΔG_r is the Gibbs free energy of the reaction ($kJ\ mol^{-1}$). The Gibbs free energy of the reaction is calculated using,

$$\Delta G_r = \Delta G_r^0 + R_g T ln Q_r \tag{A.8}$$

Here, ΔG_r^0 is the standard Gibbs free energy of the reaction ($kJ\ mol^{-1}$) that depends on the local temperature and pressure as calculated using the CHNOSZ R package [*Dick*, 2008]. R_g is the gas constant (8.3145 $J\ K^{-1}\ mol^{-1}$), T is bottom water Temperature (K) and Q_r is the reaction quotient (unitless).

Ft is the dimensionless function that varies between 0 (complete kinetic/thermodynamic limitation) and 1 (no kinetic/thermodynamic limitation),

$$F_t = \left(e^{\frac{\Delta G_r + F\Delta\Psi}{RT}} + 1\right)^{-1} \tag{A.9}$$

where, F is the Faraday constant (96.485 $kJ\ V^{-1}$), $\Delta\Psi$ is the electric potential across the membrane (0.12 mV). Both constants are taken from *Reed et al.* [2014].

A.2.2 Model parameterization

Because of a lack of reliable information, maximum rate constants of R27-R32 (Table A.6) were calibrated to geochemical depth profiles and measured rate depth profiles. The cell mass used in equation 1 (main paper) is 5 * 10^{-13} $g\ cell^{-1}$, similar as in *Louca et al.* [2016].

Cell death rates (q_r) are constant and are related to their maximum growth rate (Table A.7). The slower the cells grow the lower the death rate because the death rate is in a quasi-steady state with the growth rate [*Jørgensen and Marshall*, 2016] and are in the same range as in *Louca et al.* [2016] (Table A.7). Half saturation constants were taken from literature where possible and otherwise model constrained (Table A.7).

A.2.3 Transient modeling scenario

The model was run to steady state in 200 years. Subsequently, temporal changes were implemented that follow the periodic pulses of organic matter, Fe oxides and Mn oxides that occur ca. every 20 years [Fig. A.3 *Lenstra et al.*, 2018]. The major macrofauna species in the area are the mollusk *Limecola balthica*, the amphipod *Monoporeia affenis*, the spionid *Marenzelleria* spp. and the isopod *Saduria entomon*. *Marenzelleria* first appeared in 1995, but only became abundant after 2003, with densities at our study site ranging from 187 to 780 ind. m^{-2} [*Kauppi et al.*, 2015]. Therefore, the bioirrigation function (Fig. A.1) was only implemented in the model from 2003 onward [*Lenstra et al.*, 2018].

The transient scenario as described above (i.e. our baseline scenario) is subsequently used in a sensitivity analysis. In this analysis, the effects of variations in single parameters on the system were evaluated by running the scenario again but in this case changing (1) the bottom water salinity (0-25); (2) the bottom water O_2 concentration (0-275 $\mu mol\ L^{-1}$); (3) the organic matter deposition (factor

0.01-2) and (4) the Fe and Mn oxide deposition (factor 0.5-2) from the original values. We note that organic matter in our model consists of both carbon and nitrogen (Table A.3).

A.3 Discussion of CH_4 production and SRR

CH_4 production rates

Methanogenesis rates below the SMTZ determined in the laboratory were much higher than modeled rates in our RTM (Table A.8). There are three likely explanations for this difference. Firstly, mixing of sediments in slurry incubations can lead to higher reaction rates because of easier access of microbes to substrates than is the case in-situ. Secondly, the rate measurements were not performed at in-situ temperature. While the bottom water temperature was ca. 2.8°C, the methanogenesis rates were determined at room temperature, i.e. at ca. 21 °C. This likely led to enhanced rates of microbial activity, including CH_4 production. Thirdly, methanogenesis rates were determined ca. 1 month after sampling. During sample storage, other electron acceptors used in organic matter degradation, such as Fe and Mn oxides, which are known to be present at depth in these sediments, may have been lost, allowing methanogenesis to take over. For a detailed discussion of the problems with determining methanogenesis rates we refer to section 7 in *Reeburgh* [2007].

SO_4^{2-} reduction rates

Sulfate reduction rates (SRR) are often determined by injecting radiolabelled $^{35}SO_4^{2-}$ into sediment samples. There are however several reasons why the determined SRR, especially below the SMTZ, might not reflect actual in-situ SRR. Firstly, cryptic sulfur cycling, where sulfide is oxidized by Fe oxides, forms SO_4^{2-} and is subsequently reduced [*Holmkvist et al.*, 2011], is not included in the model. In our model, SO_4^{2-} is depleted below the SMTZ and SO_4^{2-} reduction is no longer possible below the SMTZ. However, some trace amounts of SO_4^{2-} might form via this cryptic S cycle, which subsequently can enhance SO_4^{2-} reduction below the SMTZ. If the measurements would indeed reflect the actual in-situ SRR, the difference between our model result and the measurements could be used to quantify the rate of SO_4^{2-} production via cryptic S cycling. Secondly sediment below the SMTZ

might become contaminated with $SO_4{}^{2-}$ during coring. Previous work has shown that this can lead to a slight overestimation of the $SO_4{}^{2-}$ concentration at depth [*Pellerin et al.*, 2018]. The low $SO_4{}^{2-}$ concentrations can lead to an overestimation of the SRR. For a detailed discussion of the problems with SRR measurements and their comparison with modeled SRR we refer to section 3 in *Jørgensen* [2021].

A.4 The sensitivity of changes in maximum growth rate and death rate

The model is very sensitive to changes in the maximum growth rate of the microorganisms (varied between 0.5 and 1.5 times the values in the baseline scenario). Porewater depth profiles of $SO_4{}^{2-}$, H_2S, dissolved Fe and Mn and microbial abundance quickly deviate from the baseline scenario when the growth rate is either increased or decreased (Fig. A.4). This strongly indicates that the maximum growth rates that are applied in this model are reasonable values for our system because otherwise the model would not be able to fit the measured data.

The microbial abundance of O_2 cells increases when the maximum growth rate of all microorganisms is lower because less CH_4 is oxidized by other pathways. The growth rate of O_2 cells is still relatively high and therefore the cells are able to adjust to the larger supply of CH_4 into the oxic zone of the sediment. The abundance of $SO_4{}^{2-}$-ANME strongly decreases upon a decreased maximum growth rate. As a consequence, $SO_4{}^{2-}$ penetrates deeper into the sediment because less $SO_4{}^{2-}$ is consumed by CH_4. When the maximum growth rate increases, the SMTZ is located relatively close to the sediment water interface. We find that for FeOx- and MnOx-ANMEs there is an optimum maximum growth rate, which is similar to the baseline scenario, where their abundances are high.

We found that the our model is not very sensitive to changes in the death rate of the microorganisms. The porewater depth profiles and microbial abundances only vary slightly when increasing or decreasing the death rate (Fig. A.5). We found that the abundance of the microorganisms in the zone where both the electron acceptor (i.e. O_2, $SO_4{}^{2-}$ and Fe and Mn oxides) and CH_4 are present does not change to a large extent. However, when the electron acceptor is no longer available the microorganisms die more quickly. This indicates that the growth rate is a more important regulator of microbial

abundance while the death rate becomes more important when the growth of microorganisms is limited because of substrate limitation.

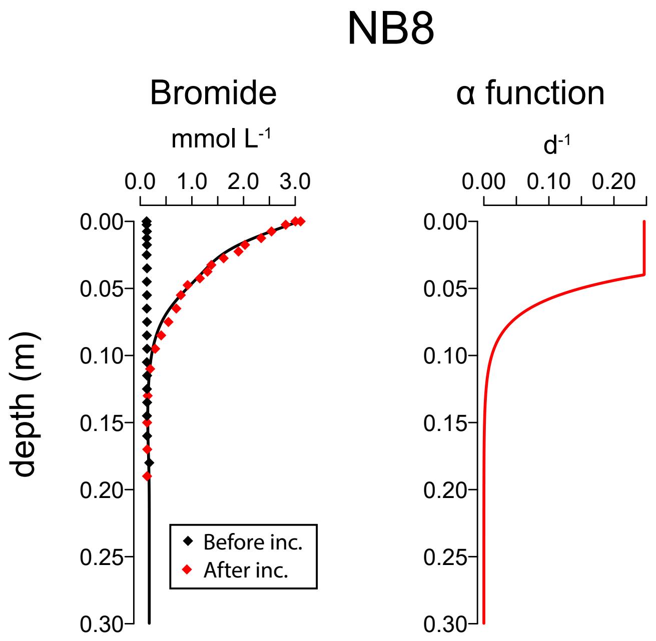

Figure A.1: Results of bromide tracer incubations at station NB8. Black and red diamonds indicate porewater bromide concentrations prior to (t=0) and after incubation, respectively. The black line indicates modeled porewater bromide concentrations, with diffusion and bioirrigation (α function).

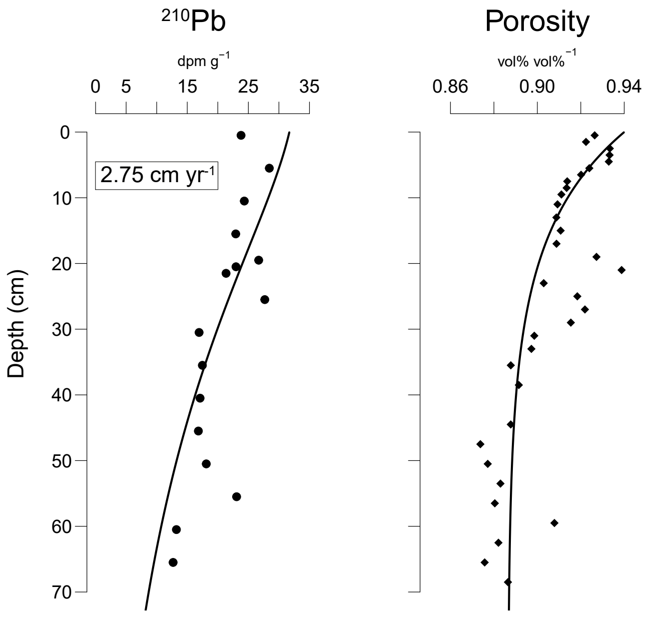

Figure A.2: Depth profiles of ^{210}Pb and porosity for sediment sampled in August 2015. Black lines are modeled depth profiles. The sedimentation rate based on the ^{210}Pb data is 2.75 $cm\ yr^{-1}$.

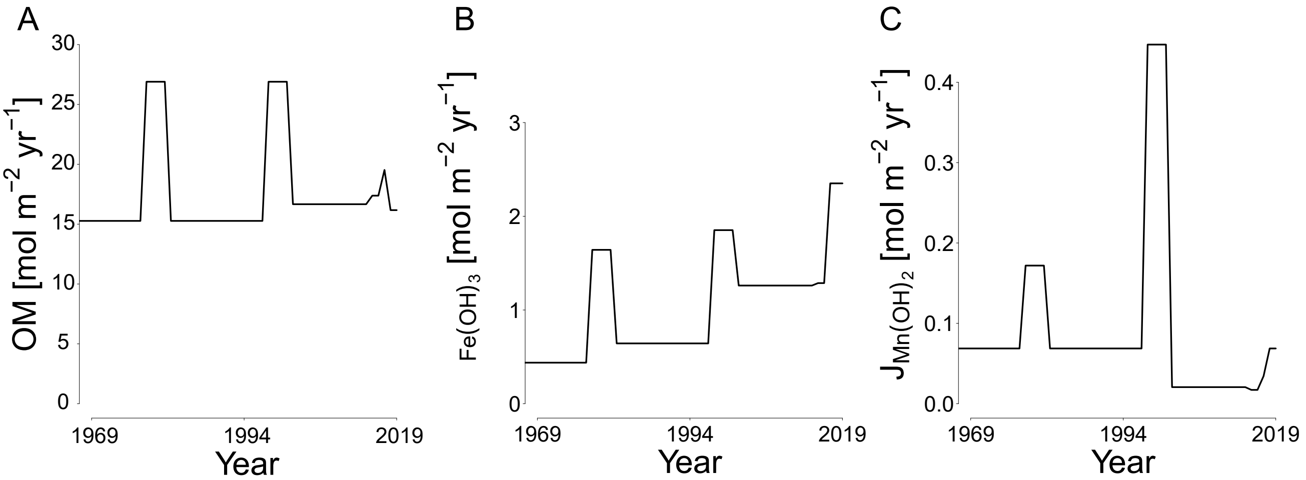

Figure A.3: Transient fluxes at the sediment–water interface from 1969 to 2019 as applied in the reactive transport model; (a) organic matter input; (b) reactive Fe oxide input; (c) reactive Mn oxide input.

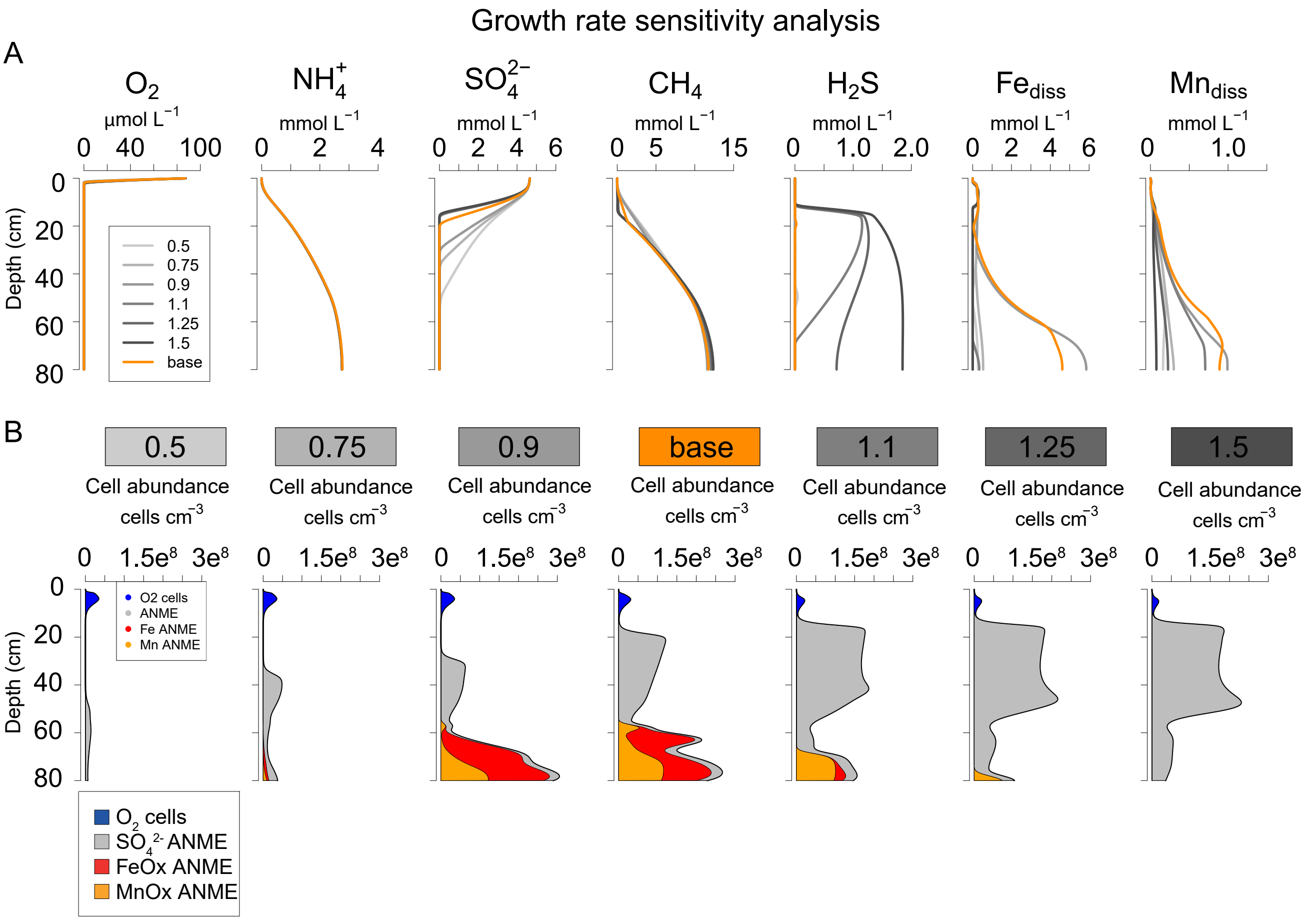

Figure A.4: Sensitivity analysis where we varied the maximum growth rate for all microorganisms with different factors (i.e. 0.5; 0.75; 0.9; 1.1; 1.25 and 1.5). (A) Porewater depth profiles for the different sensitivity analysis. (B) Cell abundances related to porewater depth profiles in figure A. The baseline scenario (orange) is fitted to the measured data and is the same as in Fig. 2.

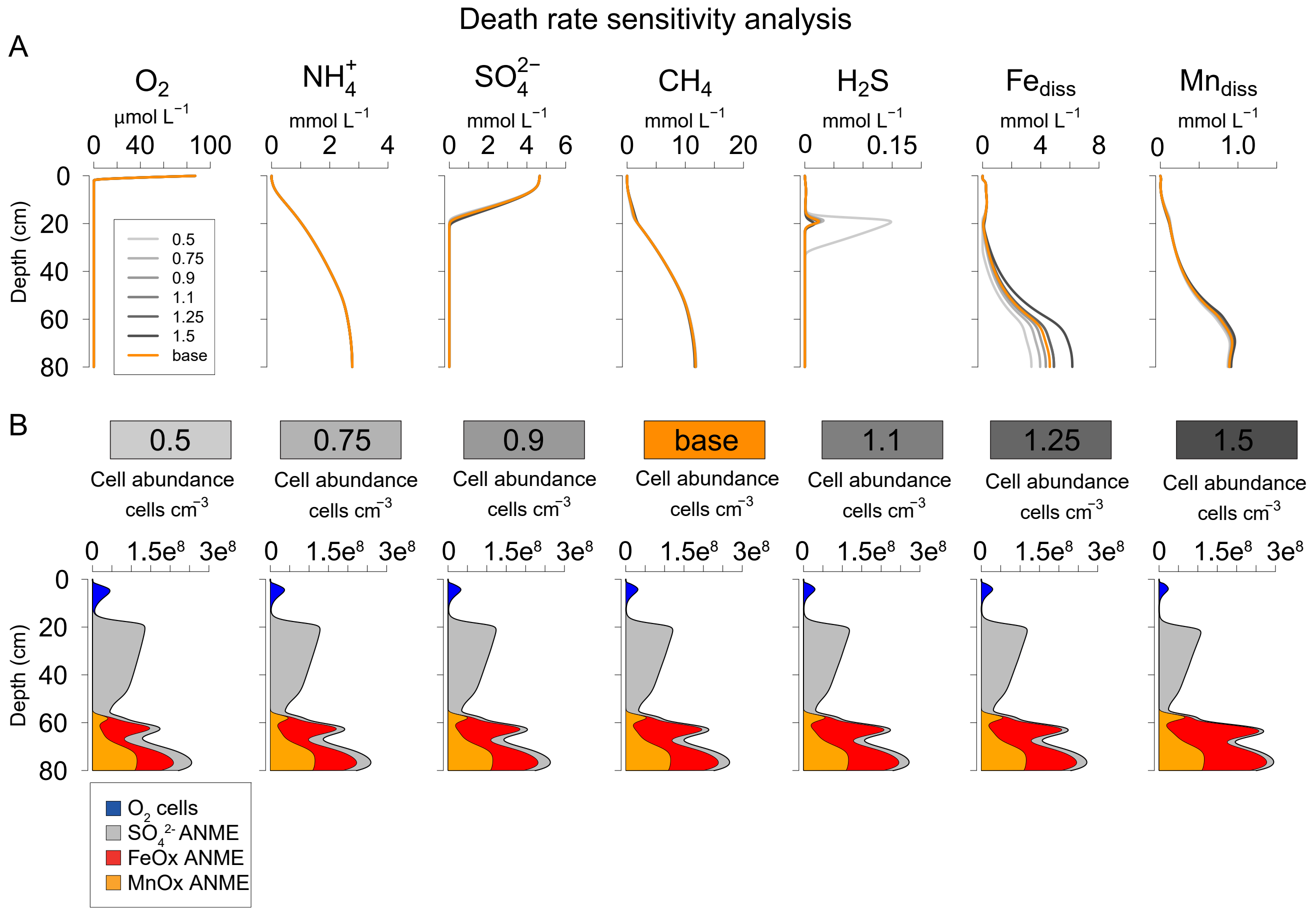

Figure A.5: Sensitivity analysis where we varied the death rate for all microorganisms with different factors (i.e. 0.5; 0.75; 0.9; 1.1; 1.25 and 1.5). (A) Porewater depth profiles for the different sensitivity analysis. (B) Cell abundances related to porewater depth profiles in figure A. The baseline scenario (orange) is fitted to the measured data and is the same as in Fig. 2.

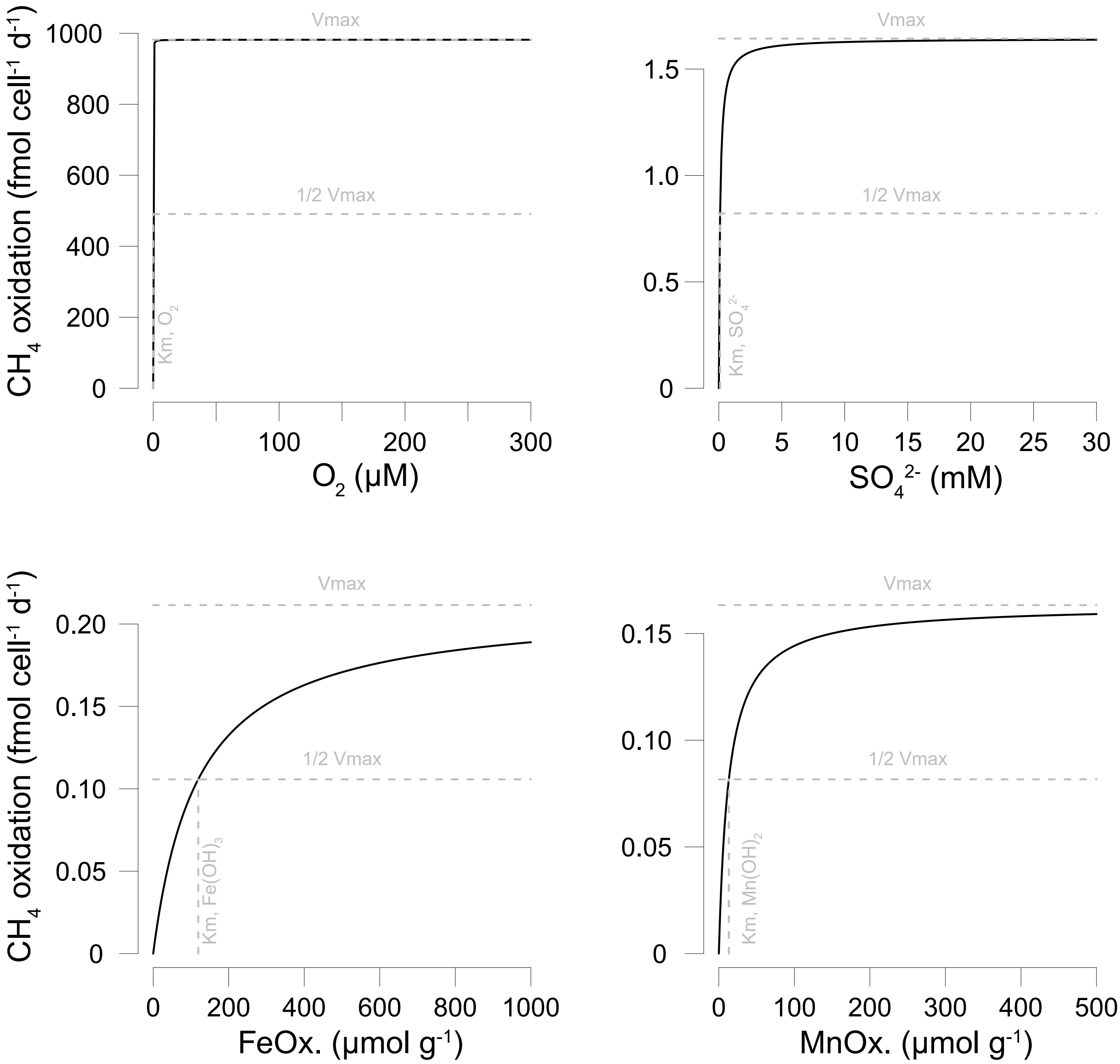

Figure A.6: Modeled methane oxidation rates ($fmol\ cell^{-1}\ d^{-1}$) plotted against substrate concentrations of O_2, SO_4^{2-}, Fe oxide and Mn oxide concentrations. Values of half rate constants included in the RTM are given in Table A.7.

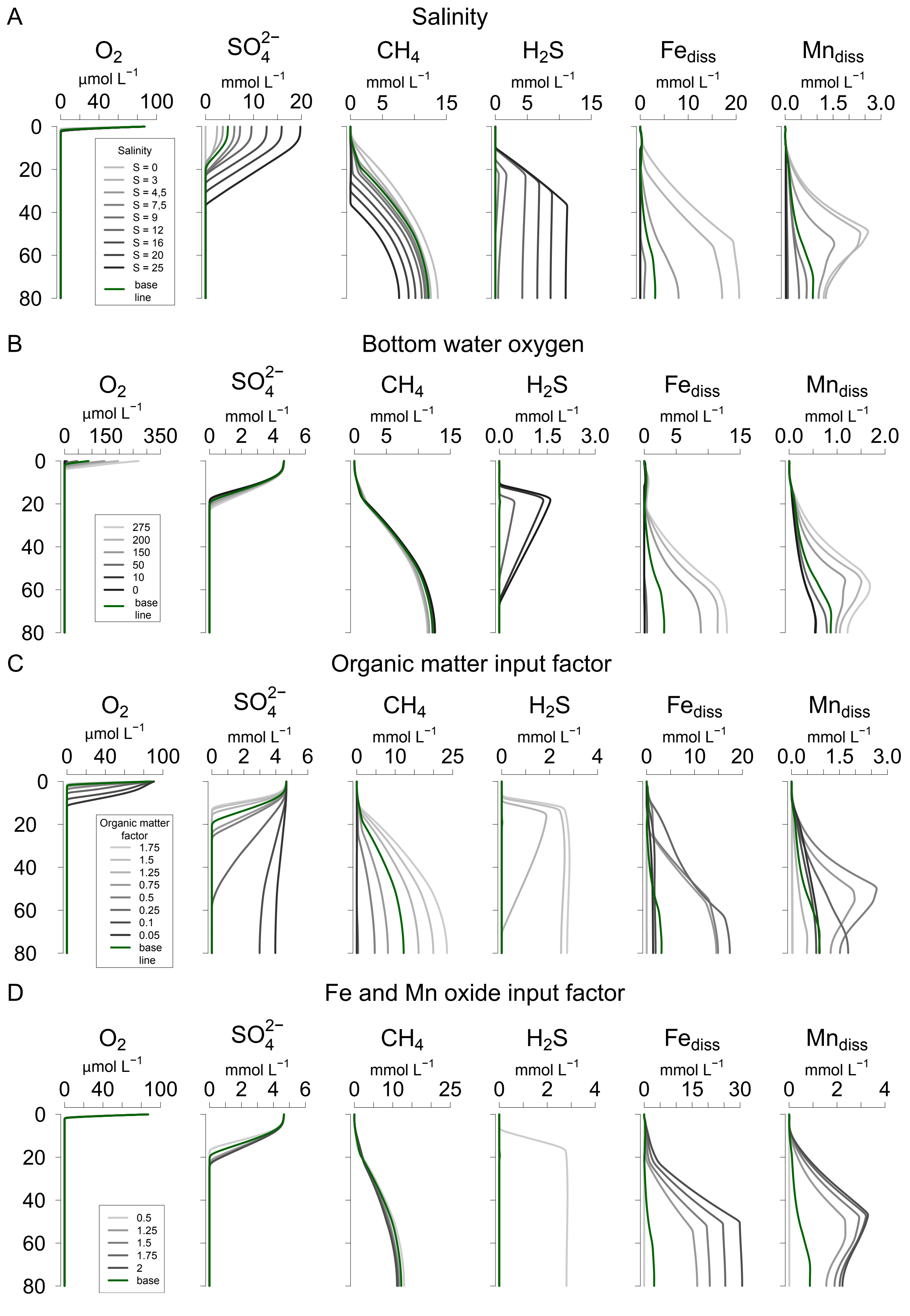

Figure A.7: Porewater profiles of O_2, SO_4^{2-}, CH_4, H_2S, dissolved Fe and dissolved Mn for the sensitivity analysis in which the following variables were varied (A) salinity; (B) bottom water O_2; (C) organic matter input factor and (D) Fe and Mn oxide input factor.

Tables Appendix

Table A.1: Sequential extraction procedure for Fe and Mn as performed on freeze-dried sediment based on *Raiswell et al.* [2010], *Claff et al.* [2010] and *Lenstra et al.* [2021].

Step	Extractant	Time (hours)	Target Fe phase	Target Mn phase
1	0.17 M sodium citrate, 0.6 M sodium bicarbonate<br>and 0.057 M ascorbic acid (pH 7.5)	24	Ferrihydrite<br>Vivianite	Easily reducible Mn oxides<br>Vivianite
2	1 M HCl	4	Easily reducible Fe oxides<br>Fe carbonates and FeS	Mn carbonates
3	50 g L^{-1} sodium dithionite solution buffered to pH 4.8 with<br>0.35 M acetic acid / 0.2 M sodium citrate	4	Crystalline Fe oxides<br>Fe in clay	Crystalline Mn oxides<br>Mn in clay
4	0.2 M ammonium oxalate/ 0.17 M oxalic acid (pH 3.2)	6	Magnetite	Mn in clay
5	70% HNO_3	2	pyrite	Mn associated pyrite

Table A.2: Chemical species and microbes included in the model.

**Species**	**Notation**
Solid	
Organic matter[a]	$OM^{\alpha,\beta,\gamma}$
Iron oxide[a]	$Fe(OH)_3{}^{\alpha,\beta,\gamma}$
Elemental sulfur	S^0
Iron monosulfide	FeS
Pyrite	FeS_2
Siderite	$FeCO_3$
Manganese oxide[b]	$MnO_2{}^{\alpha,\beta}$
Manganese carbonate	$MnCO_3$
Solute	
Oxygen	O_2
Nitrate	$NO_3{}^-$
Sulfate	$SO_4{}^{2-}$
Methane	CH_4
Iron	Fe_{diss}
Ammonium[c]	$\sum NH_4{}^+$
Hydrogen sulfide[c]	$\sum H_2S$
Dissolved Inorganic Carbon	DIC
Manganese	Mn_{diss}
Microbes	
O_2 + CH_4 cells	$\Gamma_{r,(O_2)}$
$SO_4{}^{2-}$ + CH_4 cells	$\Gamma_{r,(SO_4{}^{2-})}$
FeOx + CH_4 cells	$\Gamma_{r,(Fe(OH)_3)}$
MnOx + CH_4 cells	$\Gamma_{r,(Mn(OH)_2)}$

[a] chemical species consist of 3 types: reactive (α), less reactive (β) and refractory (γ). [b] chemical species consist of two types: reactive (α) and less reactive (β). [c] $\sum$ denotes that all species of an acid are included.

Table A.3: Reaction pathways and stoichiometries implemented in the model.

**Primary redox reactions**	
$OM^{\alpha,\beta} + aO_2 \rightarrow aCO_2 + bNH_4^+ + aH_2O$	R1
$OM^{\alpha,\beta} + 0.8aNO_3^- + 0.8aH^+ \rightarrow aCO_2 + bNH_4^+ + 0.4aN_2 + 1.4a\ H_2O$	R2
$OM^{\alpha,\beta} + 2aMnO_2^{\alpha} + 4aH^+ \rightarrow 2\ Mn^{2+} + aCO_2 + 2aH_2O$	R3
$OM^{\alpha,\beta} + 4a\ Fe(OH)_3^{\alpha} + 12aH^+ \rightarrow aCO_2 + bNH_4^+\ 13aH_2O + 4aFe_2^+$	R4
$OM^{\alpha,\beta} + 0.5a\ SO_4^{2-} + aH^+ \rightarrow aCO_2 + bNH_4^+ + 0.5aH_2S + aH_2O$	R5
$OM^{\alpha,\beta} \rightarrow 0.5aCO_2 + bNH_4^+ + 0.5aCH_4$	R6
$CO_2 + 4H_2 \rightarrow CH_4 + 2H_2O$	R7
**Secondary and other reactions**	
$2O_2 + NH_4^+ + 2HCO_3^- \rightarrow NO_3^- + 2CO_2 + 3H_2O$	R8
$O_2 + 4Fe^{2+} + 8HCO_3^- + 2H_2O + \rightarrow 4\ Fe(OH)_3^{\alpha} + 8CO_2$	R9
$2O_2 + FeS \rightarrow SO_4^{2-} + Fe^{2+}$	R10
$7O_2 + 2FeS_2 + 2H_2O \rightarrow 4SO_4^{2-} + 2Fe^{2+} + 4H^+$	R11
$2O_2 + H_2S + 2HCO_3^- \rightarrow SO_4^{2-} + 2CO_2 + 2H_2O$	R12
$2Fe(OH)_3^{\alpha} + H_2S + 4CO_2 \rightarrow 2Fe^{2+} + S^0 + 4HCO_3^- + 2H_2O$	R13
$2Fe(OH)_3^{\beta} + H_2S + 4CO_2 \rightarrow 2Fe^{2+} + S^0 + 4HCO_3^- + 2H_2O$	R14
$Fe^{2+} + H_2S \rightarrow FeS + 2H^+$	R15
$FeS + H_2S \rightarrow FeS_2 + H_2$	R16
$4S_0 + 4H_2O \rightarrow 3H_2S + SO_4^{2-} + 2H^+$	R17
$FeS + S^0 \rightarrow FeS_2$	R18
$Fe^{2+} + CO_3^{2-} \rightarrow FeCO_3$	R19
$Mn^{2+} + HCO_3^- + OH^- \rightarrow MnCO_3 + H_2O$	R20
$2Mn^{2+} + O_2 \rightarrow MnO_2^{\alpha}$	R21
$MnO_2^{\alpha,\beta} + 2Fe^{2+} + 4H_2O + \rightarrow Mn^{2+} + 2Fe(OH)_3^{\alpha} + 2H^+$	R22
$MnO_2^{\alpha} + H_2S + 2H^+ \rightarrow Mn^{2+} + S_0 + 2H_2O$	R23
$MnO_2^{\beta} + H_2S + 2H^+ \rightarrow Mn^{2+} + S_0 + 2H_2O$	R24
$FeCO_3 + H_2S \rightarrow FeS + CO_2 + H_2O$	R25
$5\ CH_4 + 8\ NO_3^- + 8\ H^+ \rightarrow 5\ CO_2 + 4\ N_2 + 14\ H_2O$	R26
$2O_2 + CH_4 \rightarrow CO_2 + 2H_2O$	R27
$SO_4^{2-} + CH_4 + CO_2 \rightarrow 2HCO_3^- + H_2S$	R28
$8Fe(OH)_3^{\alpha} + CH_4 + 15H^+ \rightarrow HCO_3^- + 8Fe^{2+} + 21H_2O$	R29
$8Fe(OH)_3^{\beta} + CH_4 + 15H^+ \rightarrow HCO_3^- + 8Fe^{2+} + 21H_2O$	R30
$4MnO_2^{\alpha} + CH_4 + 7H^+ \rightarrow 4Mn^{2+} + HCO_3^- + 5H_2O$	R31
$4MnO_2^{\beta} + CH_4 + 7H^+ \rightarrow 4Mn^{2+} + HCO_3^- + 5H_2O$	R32

Organic matter is of the form $((CH_2O)_a(NH_4^+)_b$, where a=1 and b= 1/7.1. α, β, & γ describe different fractions (i.e. highly reactive, less reactive and refractory).

Table A.4: Environmental parameters used in the model.

Description	Symbol	Value or expression	unit	source
Porosity at the Surface	ϕ_0	0.97	vol vol^{-1}	a
Porosity at depth	ϕ_∞	0.87	vol vol^{-1}	a
Porosity e-folding distance	γ	20	cm	b
Sediment density	ρ	2.65	g cm^{-3}	c
Temperature	T	2.77	°C	a
Salinity	S	5.8		a
Advective velocity of solids at depth	v_∞	$\frac{F_{sed}}{\rho(1-\phi_\infty)}$		–
Bioturbation coefficient at sediment-water interface	D_b	1.76	cm^2 yr^{-1}	d
Bioirrigation coefficient at sediment-water interface	α	90	yr^{-1}	a
Mixed layer depth	ς	4	cm	b
C:N ratio of organic matter	C/N	7.1	mol mol^{-1}	d

Sources: (a) Measured; (b) Model constrained; (c) *Reed et al.* [2011b]; (d) [*Lenstra et al.*, 2018].

Table A.5: Boundary conditions of solids and solutes at the sediment-water interface in the model. Time dependent fluxes of $OM^{\alpha,\beta,\gamma}$, $Fe(OH)_3{}^{\alpha,\beta,\gamma}$ and $MnO_2{}^{\alpha,\beta}$ at the sediment-water interface are shown in figure A.3. For all chemical species a zero-gradient boundary condition was specified at the bottom of the model domain.

Solids	Flux at sediment water interface	Unit
Sed. rate	2.75	*cm yr*$^{-1}$
F FeS	0	*mol m*$^{-2}$ *yr*$^{-1}$
F FeS_2	0	*mol m*$^{-2}$ *yr*$^{-1}$
F S0	0	*mol m*$^{-2}$ *yr*$^{-1}$
F $FeCO_3$	0.27	*mol m*$^{-2}$ *yr*$^{-1}$
F $MnCO_3$	0.05	*mol m*$^{-2}$ *yr*$^{-1}$
**Solutes**	**BW concentration**	**Unit**
C O_2	91	*µmol L*$^{-1}$
C $NO_3{}^-$	0	*µmol L*$^{-1}$
C $SO_4{}^{2-}$	4.7	*mmol L*$^{-1}$
C Fe^{2+}	0	*µmol L*$^{-1}$
C Mn^{2+}	0	*µmol L*$^{-1}$
C H_2S	0	*µmol L*$^{-1}$
C $NH_4{}^+$	0	*µmol L*$^{-1}$
C DIC	1.5	*mmol L*$^{-1}$

Table A.6: Reaction equations implemented in the model.

**Primary redox reaction equations**	
$R1 = k_{\alpha,\beta} OM^{\alpha,\beta} \left(\frac{[O_2]}{K_{m,O_2}+[O_2]}\right)$	E1
$R2 = k_{\alpha,\beta} OM^{\alpha,\beta} \left(\frac{[NO_3^-]}{K_{m,NO_3^-}+[NO_3^-]}\right)\left(\frac{K_{m,O_2}}{K_{m,O_2}+[O_2]}\right)$	E2
$R3 = k_{\alpha,\beta} OM^{\alpha,\beta} \left(\frac{[MnO_2]}{K_{m,MnO_2}+[MnO_2]}\right)\left(\frac{K_{m,NO_3^-}}{K_{m,NO_3^-}+[NO_3^-]}\right)\left(\frac{K_{m,O_2}}{K_{m,O_2}+[O_2]}\right)$	E3
$R4 = k_{\alpha,\beta} OM^{\alpha,\beta} \left(\frac{[Fe(OH)_3]}{K_{m,Fe(OH)_3}+[Fe(OH)_3]}\right)\left(\frac{K_{m,MnO_2}}{K_{m,MnO_2}+[MnO_2]}\right)\left(\frac{K_{m,NO_3^-}}{K_{m,NO_3^-}+[NO_3^-]}\right)\left(\frac{K_{m,O_2}}{K_{m,O_2}+[O_2]}\right)$	E4
$R5 = k_{\alpha,\beta} OM^{\alpha,\beta} \left(\frac{[SO_4^{2-}]}{K_{m,SO_4^{2-}}+[SO_4^{2-}]}\right)\left(\frac{K_{m,Fe(OH)_3}}{K_{m,Fe(OH)_3}+[Fe(OH)_3]}\right)\left(\frac{K_{m,MnO_2}}{K_{m,MnO_2}+[MnO_2]}\right)\left(\frac{K_{m,NO_3^-}}{K_{m,NO_3^-}+[NO_3^-]}\right)\left(\frac{K_{m,O_2}}{K_{m,O_2}+[O_2]}\right)$	E5
$R6 = k_{\alpha,\beta} OM^{\alpha,\beta} \left(\frac{K_{m,SO_4^{2-}}}{K_{m,SO_4^{2-}}+[SO_4^{2-}]}\right)\left(\frac{K_{m,Fe(OH)_3}}{K_{m,Fe(OH)_3}+[Fe(OH)_3]}\right)\left(\frac{K_{m,MnO_2}}{K_{m,MnO_2}+[MnO_2]}\right)\left(\frac{K_{m,NO_3^-}}{K_{m,NO_3^-}+[NO_3^-]}\right)\left(\frac{K_{m,O_2}}{K_{m,O_2}+[O_2]}\right)$	E6
$R7 = k_1 DIC \left(\frac{K_{m,SO_4^{2-}}}{K_{m,SO_4^{2-}}+[SO_4^{2-}]}\right)\left(\frac{K_{m,Fe(OH)_3}}{K_{m,Fe(OH)_3}+[Fe(OH)_3]}\right)\left(\frac{K_{m,MnO_2}}{K_{m,MnO_2}+[MnO_2]}\right)\left(\frac{K_{m,NO_3^-}}{K_{m,NO_3^-}+[NO_3^-]}\right)\left(\frac{K_{m,O_2}}{K_{m,O_2}+[O_2]}\right)$	E7
**Secondary redox and other reaction equations**	
$R8 = k_2[O_2][NH_4^+]$	E8
$R9 = k_3[O_2][Fe^{2+}]$	E9
$R10 = k_4[O_2][FeS]$	E10
$R11 = k_5[O_2][FeS_2]$	E11
$R12 = k_6[O_2][\sum H_2S]$	E12
$R13 = k_7[Fe(OH)_3^{\alpha}][\sum H_2S]$	E13
$R14 = k_8[Fe(OH)_3^{\beta}][\sum H_2S]$	E14
$R15 = k_9[Fe^{2+}][\sum H_2S]$	E15
$R16 = k_{10}[FeS][\sum H_2S]$	E16
$R17 = k_{11}[S^0]$	E17
$R18 = k_{12}[Fe^{2+}][S^0]$	E18
$R19 = k_{13}[Fe^{2+}][HCO_3^-]$	E19
$R20 = k_{14}[Mn^{2+}][HCO_3^-]$	E20
$R21 = k_{15}[Mn^{2+}][O_2]$	E21
$R22 = k_{16}[MnO_2^{\alpha,\beta}][Fe^{2+}]$	E22
$R23 = k_{17}[MnO_2^{\alpha}][\sum H_2S]$	E23
$R24 = k_{18}[MnO_2^{\beta}][\sum H_2S]$	E24
$R25 = k_{19}[FeCO_3][\sum H_2S]$	E25
$R26 = k_{20}[NO_3^-][CH_4]$	E26
$R27 = V_{r(O_2)} \times F_{T(O_2)} \times \frac{O_2}{K_{r,O_2}+O_2} \times \frac{CH_4}{K_{r,CH_4}+CH_4} \times \Gamma_{r,(O_2)}$	E27
$R28 = V_{r(SO_4^{2-})} \times F_{T(SO_4^{2-})} \times \frac{SO_4^{2-}}{K_{r,SO_4^{2-}}+SO_4^{2-}} \times \frac{CH_4}{K_{r,CH_4}+CH_4} \times \Gamma_{r,(SO_4^{2-})}$	E28
$R29 = V_{r(Fe(OH)_3^{\alpha})} \times F_{T(Fe(OH)_3^{\alpha})} \times \frac{Fe(OH)_3^{\alpha}}{K_{r,Fe(OH)_3^{\alpha}}+Fe(OH)_3^{\alpha}} \times \frac{CH_4}{K_{r,CH_4}+CH_4} \times \Gamma_{r,(Fe(OH)_3^{\alpha})}$	E29
$R30 = V_{r(Fe(OH)_3^{\beta})} \times F_{T(Fe(OH)_3^{\beta})} \times \frac{Fe(OH)_3^{\beta}}{K_{r,Fe(OH)_3^{\beta}}+Fe(OH)_3^{\beta}} \times \frac{CH_4}{K_{r,CH_4}+CH_4} \times \Gamma_{r,(Fe(OH)_3^{\beta})}$	E30
$R31 = V_{r(MnO_2^{\alpha})} \times F_{T(MnO_2^{\alpha})} \times \frac{MnO_2^{\alpha}}{K_{r,MnO_2^{\alpha}}+MnO_2^{\alpha}} \times \frac{CH_4}{K_{r,CH_4}+CH_4} \times \Gamma_{r,(MnO_2^{\alpha})}$	E31
$R32 = V_{r(MnO_2^{\beta})} \times F_{T(MnO_2^{\beta})} \times \frac{MnO_2^{\beta}}{K_{r,MnO_2^{\beta}}+MnO_2^{\beta}} \times \frac{CH_4}{K_{r,CH_4}+CH_4} \times \Gamma_{r,(MnO_2^{\beta})}$	E32

Table A.7: Reaction parameters used in the model.

Parameter	Value	Unit	Source	Values in literature
k_α	0.3	yr^{-1}	a,b	0.05-1.62
k_β	0.0086	yr^{-1}	b	0.0025-0.0086
K_{m,O_2}	20	$\mu mol\ L^{-1}$	c	1-30
K_{m,NO_3^-}	4	$\mu mol\ L^{-1}$	c	4-80
$K_{m,Mn(OH)_2}$	10.6	$\mu mol\ g^{-1}$	c	4-32
$K_{m,Fe(OH)_3}$	172	$\mu mol\ g^{-1}$	c	65-172
$K_{m,SO_4^{2-}}$	1.6	$mmol\ L^{-1}$	c	1.6
k1 (E7)	0.044	yr^{-1}	d	0.044
k2 (E8)	100000	$mmol\ yr^{-1}$	c,e	5000-39000
k3 (E9)	$1.4*10^5$	$mmol\ yr^{-1}$	c	$1.4*10^5$
k4 (E10)	300	$mmol\ yr^{-1}$	c	300
k5 (E11)	1	$mmol\ yr^{-1}$	c	1
k6 (E12)	160	$mmol\ yr^{-1}$	c	160
k7 (E13)	55	$mmol\ yr^{-1}$	c,f,g	8-100
k8 (E14)	44	$mmol\ yr^{-1}$	c,h	0.004-100
k9 (E15)	9000	$mmol\ yr^{-1}$	b,d	100-14800
k10 (E16)	10^{-15}	$mmol\ yr^{-1}$	d,i	10^{-15}-3.15
k11 (E17)	3	yr^{-1}	j	3
k12 (E18)	0.0025	$mmol\ yr^{-1}$	f,j	0.001-7
k13 (E19)	0	$mmol\ yr^{-1}$	i	0.0027
k14 (E20)	0.05	$mmol\ yr^{-1}$	d	0.265
k15 (E21)	1200	$mmol\ yr^{-1}$	c	800-20.000
k16 (E22)	0.002	$mmol\ yr^{-1}$	d	0.002
k17 (E23)	55	$mmol\ yr^{-1}$	d	55
k18 (E24)	44	$mmol\ yr^{-1}$	d	44
k19 (E25)	$0.4*10^{-3}$	$mmol\ yr^{-1}$	k	-
k20 (E26)	0.25	$mmol\ yr^{-1}$	k	-
K_{m,O_2}	10	$\mu mol\ L^{-1}$	l	6-32
$K_{m,Mn(OH)_2}$	13.3	$\mu mol\ g^{-1}$	k	-
$K_{m,Fe(OH)_3}$	120	$\mu mol\ g^{-1}$	k	-
$K_{m,SO_4^{2-}}$	100	$\mu mol\ L^{-1}$	m	100
K_{m,CH_4}	10	$\mu mol\ L^{-1}$	k	-
$V_{r(O_2)}$	$560*10^3$	$fmol\ cell^{-1}\ yr^{-1}$	k	-
$V_{r(Mn(OH)_2)}$	59	$fmol\ cell^{-1}\ yr^{-1}$	k	-
$V_{r(Fe(OH)_3)}$	77	$fmol\ cell^{-1}\ yr^{-1}$	k	-
$V_{r(SO_4^{2-})}$	590	$fmol\ cell^{-1}\ yr^{-1}$	k	-
*DT max. O_2 cells	<1	*days*	l	0.5-2
*DT max. SO_4^{2-}-ANME	124	*days*	n	50-210
*DT max. FeOx-ANME	203	*days*	-	-
*DT max. MnOx-ANME	163	*days*	-	-
q_{r,O_2}	3.65	yr^{-1}	k	-
$q_{r,SO_4^{2-}}$	0.0365	yr^{-1}	o	0.04-0.32
$q_{r,Fe(OH)_3}$	0.00365	yr^{-1}	k	-
$q_{r,Mn(OH)_2}$	0.00365	yr^{-1}	k	-

Sources: (a) [*Moodley et al.*, 2005]; (b) [*Reed et al.*, 2011b]; (c) [*Wang and Van Cappellen*, 1996]; (d) [*Lenstra et al.*, 2018]; (e) [*Reed et al.*, 2011a]; (f) [*Rooze et al.*, 2016]; (g) [*Egger et al.*, 2016a]; (h) [*Egger et al.*, 2016b]; (i) [*Rickard and Luther*, 1997]; (j) [*Berg et al.*, 2003]; (k) model constrained; (l) [*In 't Zandt et al.*, 2020]; (m) [*Beal et al.*, 2011]; (n) [*Knittel and Boetius*, 2009]; (o) [*Dale et al.*, 2006]; * DT max. = fastest doubling time possible in the model.

Table A.8: Potential CH_4 production rates determined for site NB8.

**Average depth (cm)**	top (cm)	bottom (cm)	CH_4 production ($\mu mol\ gsed.^{-1}\ d^{-1}$)
2	0	4	2.6
10.5	9	12	0.4
22.5	21	24	22.5
34.5	33	36	30.2
50.5	49	52	64.2
70.5	69	72	28.8

References

Aromokeye, D. A., A. C. Kulkarni, M. Elvert, and G. Wegener, Rates and microbial players of iron-driven anaerobic oxidation of methane in methanic marine sediments, *Frontiers in Microbiology*, *10*(January), 1–19, doi:10.3389/fmicb.2019.03041, 2020.

Beal, E. J., M. W. Claire, and C. H. House, High rates of anaerobic methanotrophy at low sulfate concentrations with implications for past and present methane levels, *Geobiology*, *9*(2), 131–139, doi:10.1111/j.1472-4669.2010.00267.x, 2011.

Berg, P., S. Rysgaard, and B. Thamdrup, Dynamic modeling of early diagenesis and nutrient cycling. A case study in an Arctic marine sediment, *American Journal of Science*, *303*(10), 905–955, doi:10.2475/ajs.303.10.905, 2003.

Boetius, A., et al., A marine microbial consortium apparently mediating anaerobic oxidation of methane, *Nature*, *407*(6804), 623–626, doi:10.1038/35036572, 2000.

Boudreau, B. P., On the equivalence of nonlocal and radial-diffusion models for porewater irrigation, *Journal of Marine Research*, *42*(3), 731–735, 1984.

Boudreau, B. P., The diffusive tortuosity of fine-grained unlithified sediments, *Geochimica et Cosmochimica Acta*, *60*(16), 3139–3142, doi:10.1016/0016-7037(96)00158-5, 1996a.

Boudreau, B. P., The diffusive tortuosity of fine-grained unlithified sediments, *Geochimica et Cosmochimica Acta*, *60*(16), 3139–3142, doi:10.1016/0016-7037(96)00158-5, 1996b.

Boudreau, B. P., A method-of-lines code for carbon and nutrient diagenesis in aquatic sediments, *Computers and Geosciences*, *22*(5), 479–496, doi:10.1016/0098-3004(95)00115-8, 1996c.

Boudreau, B. P., Diagenetic models and their implementation. Modelling transport and reactions in

aquatic sediments, *Springer, New York*, *505*, 436, doi:0.I007/97S-3-642-60421-S, 1997.

Burton, E. D., L. A. Sullivan, R. T. Bush, S. G. Johnston, and A. F. Keene, A simple and inexpensive chromium-reducible sulfur method for acid-sulfate soils, *Applied Geochemistry*, *23*(9), 2759–2766, doi:10.1016/j.apgeochem.2008.07.007, 2008.

Claff, S. R., L. A. Sullivan, E. D. Burton, and R. T. Bush, A sequential extraction procedure for acid sulfate soils: Partitioning of iron, *Geoderma*, *155*(3-4), 224–230, doi:10.1016/j.geoderma.2009.12.002, 2010.

Dale, A. W., P. Regnier, and P. Van Cappellen, Bioenergetic controls on anaerobic oxidation of methane (AOM) in coastal marine sediments: a theoretical analysis, *American Journal of Science*, *306*(4), 246–294, 2006.

Dick, J. M., Calculation of the relative metastabilities of proteins using the CHNOSZ software package, *Geochemical Transactions*, *9*(1), 1–17, 2008.

Egger, M., P. Kraal, T. Jilbert, F. Sulu-Gambari, C. J. Sapart, T. Röckmann, and C. P. Slomp, Anaerobic oxidation of methane alters sediment records of sulfur, iron and phosphorus in the Black Sea, *Biogeosciences*, *13*(18), 5333–5355, doi:10.5194/bg-13-5333-2016, 2016a.

Egger, M., W. Lenstra, D. Jong, F. J. Meysman, C. J. Sapart, C. Van Der Veen, T. Röckmann, S. Gonzalez, and C. P. Slomp, Rapid sediment accumulation results in high methane effluxes from coastal sediments, *PLoS ONE*, *11*(8), e0161,609, doi:10.1371/journal.pone.0161609, 2016b.

Emerson, S., R. Jahnke, and D. Heggie, Sediment-water exchange in shallow water estuarine sediments, *Journal of Marine Research*, *42*(3), 709–730, doi:10.1357/002224084788505942, 1984.

Fossing, H., and B. B. Jørgensen, Measurement of bacterial sulfate reduction in sediments: Evaluation of a single-step chromium reduction method, *Biogeochemistry*, *8*(3), 205–222, doi: 10.1007/BF00002889, 1989.

Froelich, P. N., et al., Early oxidation of organic matter in pelagic sediments of the eastern equatorial Atlantic: suboxic diagenesis, *Geochimica et Cosmochimica Acta*, *43*(7), 1075–1090, doi: 10.1016/0016-7037(79)90095-4, 1979.

Holmkvist, L., T. G. Ferdelman, and B. B. Jørgensen, A cryptic sulfur cycle driven by iron in the methane zone of marine sediment (Aarhus Bay, Denmark), *Geochimica et Cosmochimica Acta*,

75(12), 3581–3599, doi:10.1016/j.gca.2011.03.033, 2011.

In 't Zandt, M. H., A. E. De Jong, C. P. Slomp, and M. S. Jetten, The hunt for the most-wanted chemolithoautotrophic spookmicrobes, *FEMS Microbiology Ecology*, *94*(6), 1–17, doi: 10.1093/femsec/fiy064, 2020.

Jørgensen, B. B., Sulfur Biogeochemical Cycle of Marine Sediments, *Geochemical Perspectives*, *10*(2), 145–307, doi:10.7185/geochempersp.10.2, 2021.

Jørgensen, B. B., and I. P. G. Marshall, Slow microbial life in the seabed, *Annual review of marine science*, *8*, 311–332, 2016.

Kallmeyer, J., T. G. Ferdelman, A. Weber, H. Fossing, and B. B. Jørgensen, A cold chromium distillation procedure for radiolabeled sulfide applied to sulfate reduction measurements, *Limnology and Oceanography: Methods*, *2*(6), 171–180, doi:10.4319/lom.2004.2.171, 2004.

Kauppi, L., A. Norkko, and J. Norkko, Large-scale species invasion into a low-diversity system: spatial and temporal distribution of the invasive polychaetes Marenzelleria spp. in the Baltic Sea, *Biological Invasions*, *17*(7), 2055–2074, doi:10.1007/s10530-015-0860-0, 2015.

Kester, D. R., I. W. Duedall, D. N. Connors, and R. M. Pytkowicz, Preparation of artificial seawater, *Limnology and Oceanography*, *12*(1), 176–179, doi:10.4319/lo.1967.12.1.0176, 1967.

Knittel, K., and A. Boetius, Anaerobic oxidation of methane: progress with an unknown process., *Annual review of microbiology*, *63*, 311–334, doi:10.1146/annurev.micro.61.080706.093130, 2009.

Kubeneck, L. J., W. K. Lenstra, S. Y. Malkin, D. J. Conley, and C. P. Slomp, Phosphorus burial in vivianite-type minerals in methane-rich coastal sediments, *Marine Chemistry*, p. 103948, doi: 10.1016/j.marchem.2021.103948, 2021.

Lenstra, W. K., M. Egger, N. A. G. M. van Helmond, E. Kritzberg, D. J. Conley, and C. P. Slomp, Large variations in iron input to an oligotrophic Baltic Sea estuary: impact on sedimentary phosphorus burial, *Biogeosciences*, *15*(22), 6979–6996, doi:10.5194/bg-15-6979-2018, 2018.

Lenstra, W. K., R. Klomp, F. Molema, T. Behrends, and C. P. Slomp, A sequential extraction procedure for particulate manganese and its application to coastal marine sediments, *Chemical Geology*, *584*, 120,538, 2021.

Lenstra, W. K., et al., The shelf-to-basin iron shuttle in the Black Sea revisited, *Chemical Geology*,

511, 314–341, doi:10.1016/j.chemgeo.2018.10.024, 2019.

Leu, A. O., C. Cai, S. J. McIlroy, G. Southam, V. J. Orphan, Z. Yuan, S. Hu, and G. W. Tyson, Anaerobic methane oxidation coupled to manganese reduction by members of the Methanoperedenaceae, *ISME Journal*, *14*(4), 1030–1041, doi:10.1038/s41396-020-0590-x, 2020.

Louca, S., et al., Integrating biogeochemistry with multiomic sequence information in a model oxygen minimum zone, *Proceedings of the National Academy of Sciences of the United States of America*, *113*(40), E5925–E5933, doi:10.1073/pnas.1602897113, 2016.

Martin, W. R., and G. T. Banta, The measurement of sediment irrigation rates: A comparison of the Br tracer and 222-Rn/ 226-Ra disequilibrium techniques, *Journal of Marine Research*, *50*(1), 125–154, doi:10.1357/002224092784797737, 1992.

Meysman, F. J. R., B. P. Boudreau, and J. J. Middelburg, Modeling reactive transport in sediments subject to bioturbation and compaction, *Geochimica et Cosmochimica Acta*, *69*(14), 3601–3617, doi:10.1016/j.gca.2005.01.004, 2005.

Moodley, L., J. J. Middelburg, P. M. J. Herman, K. Soetaert, and G. J. de Lange, Oxygenation and organic-matter preservation in marine sediments: Direct experimental evidence from ancient organic carbon-rich deposits, *Geology*, *33*(11), 889–892, doi:10.1130/G21731.1, 2005.

Pellerin, A., G. Antler, H. Røy, A. Findlay, F. Beulig, C. Scholze, A. V. Turchyn, and B. B. Jørgensen, The sulfur cycle below the sulfate-methane transition of marine sediments, *Geochimica et Cosmochimica Acta*, *239*, 74–89, doi:10.1016/j.gca.2018.07.027, 2018.

Petzold, L., Automatic selection of methods for solving stiff and nonstiff systems of ordinary differential equations, *SIAM J Sci and Stat Comput*, *4*(1), 136–148, doi:10.1137/0904010, 1983.

Raiswell, R., H. P. Vu, L. Brinza, and L. G. Benning, The determination of labile Fe in ferrihydrite by ascorbic acid extraction: Methodology, dissolution kinetics and loss of solubility with age and de-watering, *Chemical Geology*, *278*(1-2), 70–79, doi:10.1016/j.chemgeo.2010.09.002, 2010.

Reeburgh, W., Oceanic methane biogeochemistry, *Chemical Reviews*, pp. 486–513, doi: 10.1021/cr050362v, 2007.

Reed, D. C., C. P. Slomp, and G. J. de Lange, A quantitative reconstruction of organic matter and nutrient diagenesis in Mediterranean Sea sediments over the Holocene, *Geochimica et Cosmochimica*

Acta, *75*(19), 5540–5558, doi:10.1016/j.gca.2011.07.002, 2011a.

Reed, D. C., C. P. Slomp, and B. G. Gustafsson, Sedimentary phosphorus dynamics and the evolution of bottom-water hypoxia: A coupled benthic-pelagic model of a coastal system, *Limnology and Oceanography*, *56*(3), 1075–1092, doi:10.4319/lo.2011.56.3.1075, 2011b.

Reed, D. C., C. K. Algar, J. A. Huber, and G. J. Dick, Gene-centric approach to integrating environmental genomics and biogeochemical models, *Proceedings of the National Academy of Sciences of the United States of America*, *111*(5), 1879–1884, doi:10.1073/pnas.1313713111, 2014.

Rickard, D., and G. W. Luther, Kinetics of pyrite formation by the H2S oxidation of iron (II) monosulfide in aqueous solutions between 25 and 125C: The mechanism, *Geochimica et Cosmochimica Acta*, *61*(1), 135–147, doi:10.1016/S0016-7037(96)00322-5, 1997.

Rooze, J., M. Egger, I. Tsandev, and C. P. Slomp, Iron-dependent anaerobic oxidation of methane in coastal surface sediments: Potential controls and impact, *Limnology and Oceanography*, *61*(1), doi:10.1002/lno.10275, 2016.

Rothe, M., A. Kleeberg, and M. Hupfer, The occurrence, identification and environmental relevance of vivianite in waterlogged soils and aquatic sediments, doi:10.1016/j.earscirev.2016.04.008, 2016.

Soetaert, K., and F. Meysman, Reactive transport in aquatic ecosystems: Rapid model prototyping in the open source software R, *Environmental Modelling and Software*, *32*, 49–60, doi: 10.1016/j.envsoft.2011.08.011, 2012.

Soetaert, K., T. Petzoldt, and F. J. R. Meysman, Marelac: Tools for Aquatic Sciences v2.1.3, *R package*, 2010.

Wang, Y. F., and P. Van Cappellen, A multicomponent reactive transport model of early diagenesis: Application to redox cycling in coastal marine sediments, *Geochimica Et Cosmochimica Acta*, *60*(16), 2993–3014, doi:10.1016/0016-7037(96)00140-8, 1996.
